# Beta-Glucans Supplementation Associates with Reduction in P-Cresyl Sulfate Levels and Improved Endothelial Vascular Reactivity in Healthy Individuals

**DOI:** 10.1371/journal.pone.0169635

**Published:** 2017-01-20

**Authors:** Carmela Cosola, Maria De Angelis, Maria Teresa Rocchetti, Eustacchio Montemurno, Valentina Maranzano, Giuseppe Dalfino, Carlo Manno, Annapaola Zito, Michele Gesualdo, Marco Matteo Ciccone, Marco Gobbetti, Loreto Gesualdo

**Affiliations:** 1 Department of Emergency and Organ Transplantation – Nephrology, Dialysis and Transplantation Unit, University of Bari Aldo Moro, Bari, Italy; 2 Department of Soil, Plant and Food Sciences, University of Bari Aldo Moro, Bari, Italy; 3 Department of Emergency and Organ Transplantation - Cardiovascular Disease Unit, University of Bari Aldo Moro, Bari, Italy; 4 Faculty of Science and Technology, Free University of Bozen, Bolzano, Italy; Universita degli Studi di Milano, ITALY

## Abstract

**Background:**

Oat and barley beta-glucans are prebiotic fibers known for their cholesterol-lowering activity, but their action on the human gut microbiota metabolism is still under research. Although the induction of short-chain fatty acids (SCFA) following their ingestion has previously been reported, no study has investigated their effects on proteolytic uremic toxins p-cresyl sulfate (pCS) and indoxyl sulfate (IS) levels, while others have failed to demonstrate an effect on the endothelial function measured through flow-mediated dilation (FMD).

**Objective:**

The aim of our study was to evaluate whether a nutritional intervention with a functional pasta enriched with beta-glucans could promote a saccharolytic shift on the gut microbial metabolism and improve FMD.

**Methods:**

We carried out a pilot study on 26 healthy volunteers who underwent a 2-month dietary treatment including a daily administration of Granoro “Cuore Mio” pasta enriched with barley beta-glucans (3g/100g). Blood and urine routine parameters, serum pCS/IS and FMD were evaluated before and after the dietary treatment.

**Results:**

The nutritional treatment significantly reduced LDL and total cholesterol, as expected. Moreover, following beta-glucans supplementation we observed a reduction of serum pCS levels and an increase of FMD, while IS serum levels remained unchanged.

**Conclusions:**

We demonstrated that a beta-glucans dietary intervention in healthy volunteers correlates with a saccharolytic shift on the gut microbiota metabolism, as suggested by the decrease of pCS and the increase of SCFA, and associates with an improved endothelial reactivity. Our pilot study suggests, in addition to cholesterol, novel pCS-lowering properties of beta-glucans, worthy to be confirmed in large-scale trials and particularly in contexts where the reduction of the microbial-derived uremic toxin pCS is of critical importance, such as in chronic kidney disease.

## Introduction

A huge amount of scientific evidence is shedding new light on the relationship, both in healthy and in disease conditions, between the human body and its symbiotic super-organism hosted in the intestine: the microbiota [1]. It is by now established that, in the complex functioning of the human organism, it plays a central role [2]. Beyond its contribution to physiological and “beneficial” functions such as the development of the immune system and the energy-harvesting from indigestible complex carbohydrates, the role of microbiota in a variety of diseases is gradually emerging: chronic kidney disease (CKD), obesity, diabetes and cardiovascular disease (CVD) show the presence of a dysregulation (so-called “dysbiosis”) of the gut microbiota composition and metabolism [3–6]. Microbial metabolism represents the molecular link by which microbiota interacts with human physiology and diseases [7]. This metabolism, mainly divided in saccharolytic or proteolytic, is generally believed to foster health when the balance is shifted towards the first one because of the different actions of the metabolic products derived from the two catabolic pathways [8]. The saccharolytic one, in fact, mainly leads to the release of short-chain fatty acids (SCFA), known for their immune-modulating, anti-inflammatory and in general beneficial activities [9–12]. Conversely, the downstream metabolites of the proteolytic pathway are represented by “toxic” compounds [13], among which p-cresyl sulfate (pCS) and indoxyl sulfate (IS), normally excreted by the kidneys, but emerging as the main uremic toxins which accumulate in the blood when the kidney function declines, such as in CKD, where they are by now recognized to promote inflammation, cardiovascular complications and disease progression [14,15]. Nutritional strategies acting on the gut microbiota to restore health are appealing areas of research, since prebiotic fibers and food in general offer the unique possibility to modulate the microbiota composition and metabolism [16,17]. In fact, non-refined cereals, legumes and in general plant-derived food, such as the ones abundantly represented in Mediterranean Diet, are supposed to promote intestinal wellness (and subsequently a general healthy status) by acting a “selective pressure” on saccharolytic bacteria and metabolism [18].

Beta-glucans are dietary fibers mainly found in whole-grain cereals, such as barley and oat, already recognized for their ability to lower LDL and total cholesterol [19] in a dose of 3 daily grams. EFSA and FDA allow declaring this nutritional claim in labels of commercially available functional foods [20–23]. Some researches on humans demonstrated the increase of circulating SCFA following beta-glucans ingestion [24], and also our group recently published results showing the modulation of the gut microbiota composition and the increase of SCFA levels after beta-glucans treatment [25], but no human trial investigated, at the same time, the effects on proteolytic uremic toxins pCS and IS. Additionally, some studies were set up in order to investigate the presumptive beta-glucans effects on ameliorating the endothelial function [26–28] but again, in our knowledge, none demonstrated any effect on flow-mediated dilation (FMD).

The aim of our study was to evaluate whether a nutritional intervention with a functional pasta enriched with beta-glucans could be able to effectively reduce the proteolytic metabolism, in addition to the increase of the saccharolytic one, and to ameliorate the endothelial function, on an *in vivo* human study. To this purpose, we carried out a pilot study on 26 healthy volunteers, which underwent a 2-months dietary treatment including a daily administration of Granoro “Cuore Mio” pasta enriched with barley beta-glucans (3g/100g). Blood and urine routine parameters, serum pCS/IS and FMD were evaluated before and after the dietary treatment.

## Materials and Methods

### Patients and study design

The pilot study was carried out in accordance with the Helsinki Declaration (IV Adaptation) and the European Guidelines for Good Clinical Practice. As a pilot study, no sample size calculation was performed and a target of 30 participants was established. The protocol of the study was approved by the Ethical Committee of the Azienda Ospedaliero-Universitaria Consorziale Policlinico of Bari, Italy (Authorization nr. 1570/2014, 1^st^ December 2014). The authors confirm that all ongoing and related trials for this intervention are registered in the ClinicalTrials.gov registry database; because of administrative issues that led to a delay in the ClinicalTrials.gov registration, we registered the trial after the enrolment of the participants started (Identifier nr. NCT02710513, 7^th^ March 2016). Healthy volunteers were enrolled according to the following inclusion/exclusion criteria: (i) Inclusion criteria: Healthy people aged 30–70 years old; BMI ranged between 18.5 and 24.9; omnivorous diet; (ii) Exclusion criteria: Diabetes type 2; urine protein > 1g/24h; antibiotics and probiotics administration by 15 days before the enrollment; gastrointestinal, celiac, inflammatory systemic and chronic liver diseases; recent diagnosis of cancer; corticosteroid or immunosuppressive therapies; previous major acute cardiovascular pathologies (heart attack, cerebral ictus); hyperlipidemia; consume of alcohol; psychiatric diseases.

Primary Outcome: Reduction in LDL and total cholesterol; Secondary Outcomes: modulation of SCFA fecal levels, modulation of pCS and IS, effects on FMD. The study was carried out at the ambulatories of the Nephrology and Cardiovascular Disease Units of our Department. After a two-months run-in period in which each volunteer followed a Mediterranean-based free diet including a daily supply of 100 g of usual pasta, each volunteer (grouped in 4–6 people per day) was given a supply of pasta “Cuore Mio” Granoro (Pastificio Attilio Mastromauro Granoro s.r.l.—Corato BA–Italy) and was instructed to include a daily portion (100 g, dry weight) of functional pasta in their Mediterranean-based diet for two months. The pasta was made with mixed durum wheat (75%) and whole barley (25%) flour, providing an amount of 3 g of beta-glucans for 100 g of pasta. Each person was visited before (T0) and after (T2) the beta-glucans intervention, and anthropometric parameters (height, weight, BMI) and FMD measurements were taken and registered on an electronic case report form. Food frequency and 24-hours recall questionnaires were administered in order to obtain information about food intake, and blood and feces samples were collected. The latter were used to determine SCFA concentration, by gas-chromatography mass spectrometry-solid-phase microextraction (GC-MS/SPME), as detailed elsewhere [25]. No incentive was provided to the volunteers.

### Nutritional analysis

As previously reported [25], the FFQ and the 24-hours recall questionnaire administered at T0 and T2 were used to extrapolate the data on a single component intake, by using the official Italian food composition databases (INRAN, http://nut.entecra.it/646/tabelle_di_composizione_degli_alimenti.html and IEO, http://www.bda110ieo.it/uk/index.aspx) and to calculate the PREDIMED score [29].

### Blood analyses

Blood samples were processed for routine analyses, including metabolic parameters: total and high-density lipoprotein (HDL) cholesterol and glycaemia were measured using Siemens enzymatic methods (Dimension Vista 1500, Siemens Health Diagnostics, Deerfield, IL), while glycated haemoglobin (HbA1c) levels were determined using high-performance liquid chromatography (BioRad D10, Pratteln, Switzerland). LDL cholesterol levels were estimated by using the Friedewald equation for values < 300 mg/dl. An additional aliquot of blood for each patient/time point was centrifuged at 3000 rpm for 10 minutes. The obtained serum samples were then stored at −80°C until use.

### Liquid chromatography/electrospray ionization–mass spectrometry/mass spectrometry (LC/ESI-MS/MS) for quantification of uremic toxins pCS and IS

Serum samples were treated following the procedure reported by Itoh [30]. Briefly, unprocessed serum (20 μL) was diluted with 40 μL of distilled water. A 50 μL aliquot of diluted serum was added to 200 μL acetonitrile containing internal standard (100 ng/mL of stable-isotope-labelled compounds; indoxyl-d4 sulphate from Toronto Research Chemicals, North York, ON, Canada) in a Sirocco 96-well protein precipitation plate (Waters, Milford, MA, USA), and the mixture was mixed and centrifuged to remove protein precipitation according to the manufacturer’s protocol. After centrifugation, 40 μL of filtrate, eluted in the Sirocco’s collection plate, were diluted with 120 μL of 5 mmol/L ammonium acetate solution (Sigma) before LC/MS/MS analysis. Quantitative analysis of pCS and IS was performed by selected reaction monitoring (SRM) of LC/ESI-MS/MS. HPLC analysis of each sample (5 μL) was performed using a gradient elution with a LC-20Avp LC system (Shimadzu, Kyoto, Japan) on an Atlantis dC18 column (2.1 mm×50 mm, 3 μm) (Waters, Milford, MA, USA) at a flow rate of 0.2 mL/min with the column maintained at 40°C. The gradient solution consisted of a solvent A (5 mmol/L ammonium acetate solution) and a solvent B (methanol). We operated in a negative ion mode with an elution solution of 20% B (A:B; 80:20, by volume) for 2 min followed by a linear gradient up to 60% B for the next 2 min and up to 95% for the next 3 min. After 1.5 min at 95% B it was returned to 20% B over the next 0.5 min, followed by 20% B for 11 min, making a total cycle time of 20 min/sample.

The SRM method of LC/ESI-MS/MS was carried out using a triple quadrupole mass spectrometer (API4000, AB SCIEX, Carlsbad, CA, USA) equipped with an ESI source. The MS/MS parameters for the quantification of IS were: Q1 (212.08 m/z), Q3 (80.0 and 132.0 m/z), retention time (Rt, 2.4 min), DP (-38.40 V), EP (-11.00 V), CP (-25.80 V), CXP (-4.87 V). The MS/MS parameters for the quantification of pCS were: Q1 (186.8 m/z), Q3 (106.9 m/z), retention time (Rt, 4.66 min), DP (-50.30 V), EP (-6.30 V), CP (-31.00 V), CXP (-14.00 V). MS/MS operating conditions were as follows: curtain gas: 20 psi; ion source gas 1: 60 psi; ion source gas 2: 70 psi; collision gas: 4; ESI: -4 kV; ion source temperature: 500°C; interface heater: ON. Data acquisition and processing were carried out using the software package Analyst 1.6.2. To calibrate the total serum concentration, a 40 μL aliquot of the commercially available pCS (ALSACHIM, Bioparc ILLKIRCH, FRANCE) and IS (Toronto Research Chemicals, North York, ON, Canada) of known concentrations in distilled water (seven concentrations of each metabolite were used for calibration curve) was spiked into a 20 μL aliquot of a pool of healthy subjects human serum, which was pre-processed in active carbon (2.5 g / 50 mL serum) to remove internal metabolites. The serum containing the known aliquots of pCS and IS was then processed as described previously. Calibration curve range for both metabolites was from 0.02 to 2 μg/mL and correlation coefficients (r) for the measurement of total serum concentration was 0.9994 and 0.9999 for pCS and IS, respectively. The calibration curves showed almost linear correlation coefficients in the range between the minimum and maximum concentrations. Samples with pCS and IS levels beyond the maximum concentrations of the calibration curves were diluted with distilled water and then reanalysed by LC/ESIMS/MS analysis.

### FMD

FMD is considered as an index of nitric oxide-mediated vasodilatation. As it is known that temperature, food, stress, drugs and sympathetic stimuli influence the FMD [31], we performed the study with the subjects fasting for at least 8–12 hours, in a quiet air conditioned room (22–24°C), early in the morning. Moreover, the subjects were asked not to exercise, smoke, or take exciting substances like coffee/tea, chocolate which could impair the endothelial function and for at least 4–6 hours before the exam. The subjects were positioned supine and underwent a preliminary evaluation to explore the anatomy and identify landmarks; particular attention was directed to poor quality images, the presence of atherosclerotic plaques, calcifications, arterial tortuosity or kinking. The scan was done at the level of the right brachial artery between 5 and 10 cm above the antecubital fossa using a 7.0 MHz or higher linear probe. The study was performed using a high resolution ultrasonograph (Philips Sonos 5500) connected to an image analysis system, certified by the CNR of Pisa (MVE II) [32], for computing the brachial artery diameter in real-time by analyzing B-mode ultrasound images, setting positivity to the test value at less than 5%. All the ultrasound examinations were performed by the same physician in order to reduce the observer bias. With the subject in supine position for at least 10 minutes, the arm was positioned comfortably in such a way as to get good images of the humeral artery. The selected artery segment to be displayed was above the antecubital fossa in a long axis projection, in order to identify the part where the anterior and posterior intimal interfaces between the lumen and vessel wall were clear. Moreover, in order to maintain the same image during the whole study, we used a probe-supporting device. A sphygmomanometer cuff was placed in the distal site to the humeral artery, i.e. on the forearm, and then, the brachial artery profile was manually traced on image analysis system. After 1 minute of flow image baseline acquisition, the artery was occluded by inflating the cuff to at least 50 mmHg above systolic pressure for exactly 5 min [31]. When the cuff was deflated, it induced a short state of high flow (reactive hyperemia) through the brachial artery due to the reduced downstream resistance caused by the ischemia-induced dilatation. The resulting increased shear stress provides the stimulus for nitric oxide release from endothelium and dilatation of the humeral artery. Within 15 seconds from the end of artery occlusion, the flow velocity was measured and then the degree of hyperemia. Furthermore, the image of the artery was recorded for 3 minutes after cuff deflation. The image analysis system shows the instantaneous diameter of brachial artery throughout the study and draws the diameter curve. The latter is automatically analyzed, providing the FMD value corrected for age, gender and body weight, as the ratio of the change in diameter (difference between the maximum post-deflation and baseline value) divided by the baseline value. FMD was analyzed as the percentage increase in brachial artery diameter compared to the baseline after the application of a pressure stimulus.

### Statistical analysis and correlations

All the analyses, performed at group level, were based on the intention to treat. Experimental data are presented in tabular form as mean ± SD (parametric variables) or median and interquartile range (non-parametric variables) and in graphical form as mean ± SEM or median and interquartile range, respectively. Differences between quantitative parametric variables were analyzed by Student two-tailed, paired t-tests, while differences between quantitative nonparametric variables were tested against Wilcoxon test as appropriate. Laboratory values, pCS and FMD passed the D’Agostino&Pearson normality test, while IS did not follow a normal distribution. For this reason, the Pearson analysis was applied to the correlation between parametric variables, except that between pCS and IS, performed with the Spearman test. Statistical significance was considered when p values were < 0.05. All the analyses were performed using GraphPad Prism (GraphPad software, version 6, San Diego, CA).

## Results

### Study population

**Fig 1:**
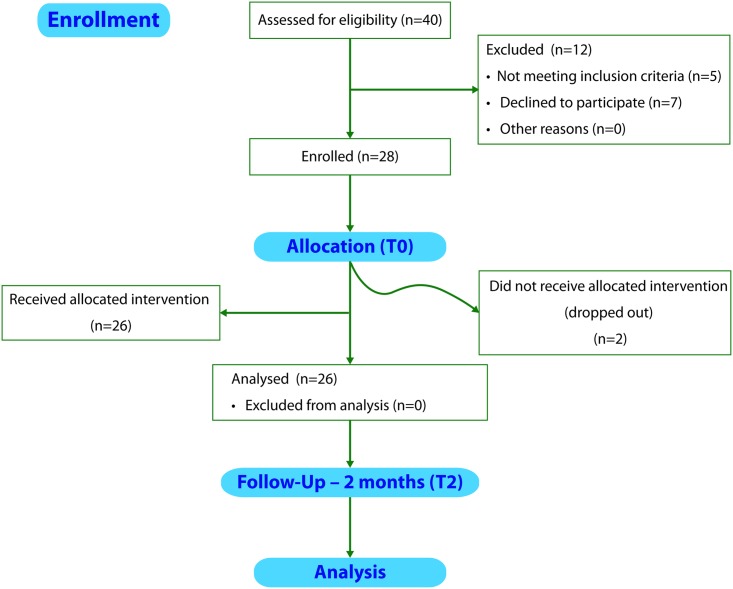
Study flow diagram. Graphical study representation, adapted from CONSORT^®^ 2010 flow diagram, showing the total number of people assessed for eligibility, enrolled, undergoing intervention and analyzed.

The study started in December 2014 and ended in April 2015. In order to ensure the target of 30 enrolled volunteers, as many as 40 people were assessed for eligibility between acquaintances of the study staff and screened after a first phone contact: 12 were excluded (5 did not meet the inclusion criteria and 7 declined to participate). As many as 28 healthy volunteers (12 males and 16 females) were recruited after they signed a written informed consent. 2 subjects (1 male, 1 female) dropped out before starting the intervention with the functional pasta for personal reasons. The remaining 26 volunteers (11 males, 15 females, Table 1) completed the 2-month intervention (Fig 1) and their data were included in the final analysis.

**Table 1:** Descriptive characteristics and study results.

	T0	T2	*p* value
**Age**	38.0 (34.5–43.0)	-	-
**HDL cholesterol (mg/dl)**	62.6 ± 16.6	62.4 ± 16.6	0.92
**LDL cholesterol (mg/dl)**	107.4 ± 25.2	93.8 ± 24.5	0.003
**Total cholesterol (mg/dl)**	183.8 ± 30.3	173.3 ± 27.4	< 0.001
**pCS (ppm)**	2.15 ± 1.22	1.48 ± 1.25	0.02
**IS (ppm)**	0.52 (0.37–0.77)	0.60 (0.44–0.73)	0.75
**FMD (%)**	7.2 ± 1.6	9.5 ± 3.3	0.002
**Glycaemia (mg/dl)**	79.96 ± 8.56	82.33 ± 7.39	0.03
**HbA1c (mmol/mol)**	31.29 ± 3.33	34.13 ± 3.37	< 0.001

The table reports the median age of the enrolled subjects and the numerical results of the study, before (T0) and after the 2-month intervention (T2). Parametrical data (HDL, LDL and total cholesterol, pCS, FMD, glycaemia, HbA1c) are represented as mean ± SD, non-parametrical ones (age, IS) are reported as median and interquartile range; p-values of t-student and Wilcoxon tests of the differences between T0 and T2 are reported, respectively. Age, LDL and total cholesterol data already published by De Angelis *et al*. [25]. Abbreviations: HDL (high-density lipoprotein), LDL (low-density lipoprotein), pCS (p-cresyl sulfate), IS (indoxyl sulfate), FMD (flow-mediated dilation), HbA1c (glycated haemoglobin).

### Diet intervention and serum levels of cholesterol

**Fig 2:**
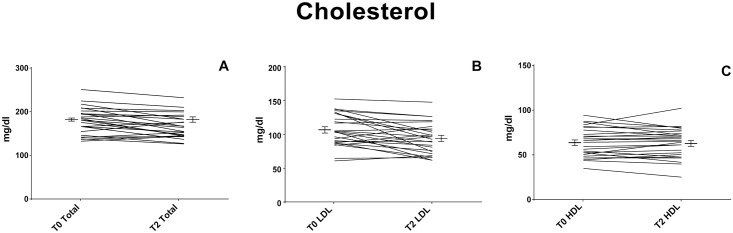
Pasta enriched with beta-glucans lowered LDL and total cholesterol serum levels. Serum levels of HDL (Fig 2a), LDL (Fig 2b) and total cholesterol (Fig 2c) of healthy subjects before (T0) and after (T2) two months of diet intervention with pasta enriched with barley β- glucans. Statistically significant difference (* p<0.001; $ p<0.05). Graphical representation of data published by De Angelis *et al*. [25].

As previously reported [25], the diet intervention with pasta enriched with barley beta-glucans (3 g for 100 g of pasta) did not change (p>0.05) the total daily intake of carbohydrates, total proteins, fat, minerals (sodium, potassium, iron, calcium and phosphorus), and vitamins (thiamine, riboflavin, niacin, Vit. C and Vit. E). The only exception was the total amount of fibers which was the highest in the volunteers after ingestion of pasta enriched with barley beta-glucans (13.4 and 22.0 before and after diet intervention respectively, p<0.001). The adherence to the Mediterranean Diet was assessed by the calculation of the PREDIMED score [29], ranging from 8.1±1.7 before the intervention to 8.2±1.7 after it, without any significant difference pre and post-intervention, confirming that the beta-glucans supplementation was the only change introduced in volunteers diet. No particular clinical condition or adverse effect was reported by any of the volunteers. In addition, the validity of the study was also confirmed by the reduction of serum levels of total cholesterol (183.8±30.3 vs 173.3±27.4 mg/dl; confidence intervals (CI) [171.0–196.6] vs [161.7–184.8]; p<0.001) and LDL cholesterol (107.4±25.2 *vs* 93.8±24.5 mg/dl; CI [96.7–118.0] vs [83.4–104.1]; p = 0.003) in the whole population (Table 1, Fig 2). No difference was observed in HDL cholesterol levels (62.6±16.6 *vs* 62.4±16.6 mg/dl; p = 0.92). Although reduced, cholesterol levels remained in the normality range.

### Serum levels of pCS and IS

**Fig 3:**
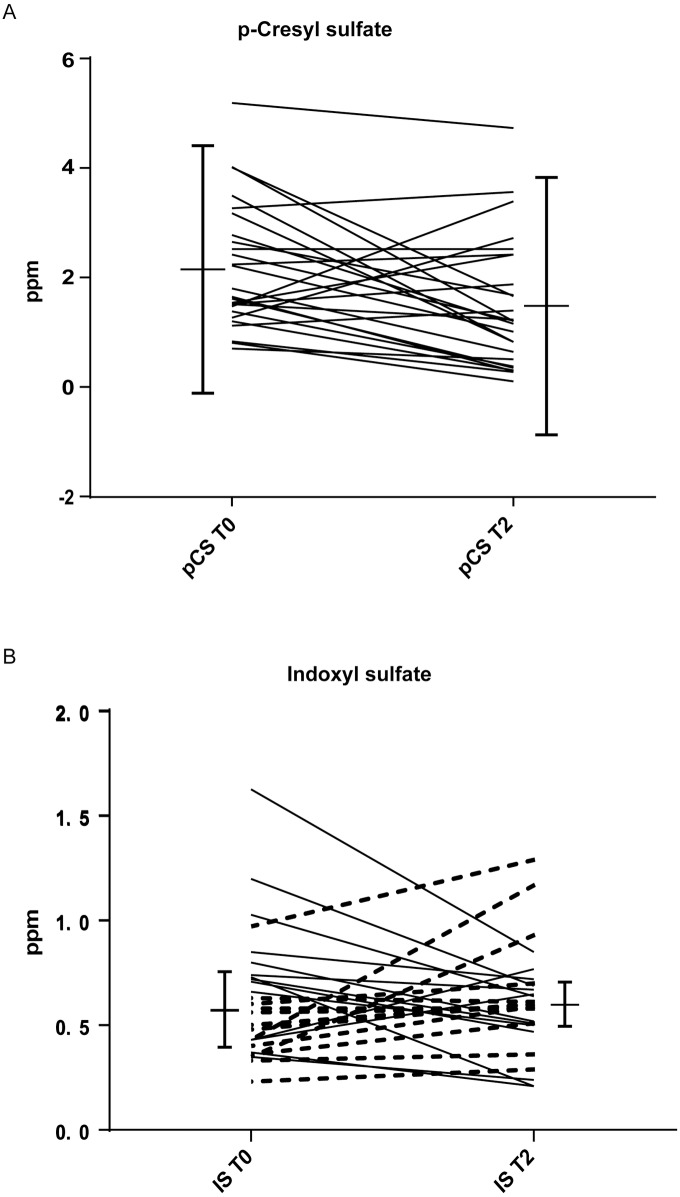
Pasta enriched with beta-glucans lowered pCS, but not IS serum levels. Serum levels of pCS (A) and IS (B) of healthy subjects before (T0) and after (T2) two months of diet intervention with pasta enriched with barley β-glucans. In Fig 3b, continuous and dotted lines represent decreased and increased IS levels after the intervention, respectively. *statistically significant difference (p<0.05).

In order to evaluate the effects of the dietary intervention on the microbiota metabolism, we performed analyses of circulating pCS and IS. We observed a significant decrease in pCS (2.15±1.22 *vs* 1.48±1.25 ppm; CI [1.6–2.7 vs 0.9–2.0]; p = 0.02) serum levels (Table 1, Fig 3a), while IS levels remained unchanged in the population as a whole (Table 1, Fig 3b).

### Improvement of vascular function

**Fig 4:**
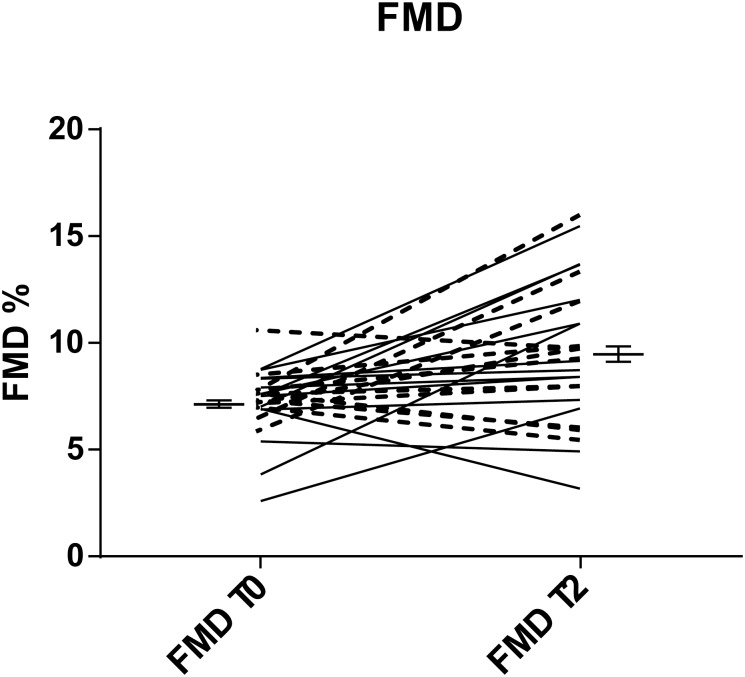
Improvement of FMD after the nutritional intervention. FMD measured before (T0) and after (T2) two months of diet intervention with pasta enriched with barley β-glucans. Gender is evidenced with continuous and dotted lines for females and males, respectively. *statistically significant difference (p<0.05).

We measured endothelial function by FMD before and after the 2-month intervention period. In order to avoid biases in the results, we took measurement at the same time in the morning. We observed a marked and significant increase in the FMD value (7.20±1.58 vs 9.47±3.34; CI [6.6–7.8] vs [8.1–10.8]; p = 0.002) (Fig 4), which indicated an improved vascular function.

### Correlations

**Fig 5:**
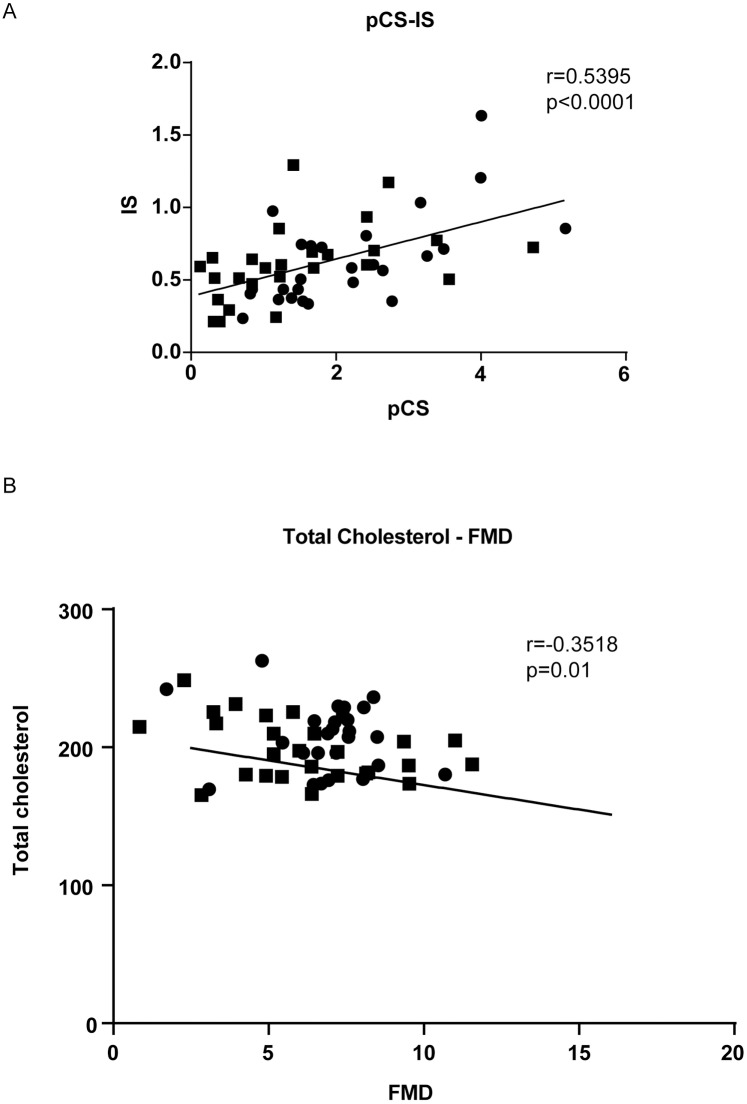
pCS-IS and total cholesterol-FMD correlations. Graphical representation of the direct correlation between pCS and IS (Fig 5a) and of the inverse correlation between total cholesterol and FMD (Fig 5b). Round and squared points represents T0 and T2 values, respectively. Correlation coefficients and p values are represented in the figure.

Consistently with their common biosynthetic pathway derived from the proteolytic metabolism, we found a positive correlation between pCS and IS (Fig 5a). Moreover, we evidenced an inverse correlation between FMD and total cholesterol (Fig 5b).

## Discussion

In this study we demonstrate that a two-month dietary treatment providing a daily supply of 3g of beta-glucans, beyond its well-known cholesterol-lowering action, is associated with a saccharolytic shift in the gut microbiota metabolism and an improvement of the endothelial function, in a cohort of healthy volunteers. The dietary intervention, in fact, modulated microbial metabolic markers panel, by decreasing pCS serum levels and increasing fecal SCFA concentration, and was associated with an improved FMD.

Beta-glucans are fibres known for their ability to reduce LDL and total cholesterol [19–23]. The aim of the present study was to explore additional health properties of beta-glucans. In particular, we focused our attention on their action on the gut microbiota metabolism by analysing systemic and local metabolic markers, and on the endothelial function through the evaluation of FMD. Beyond the already reported reduction of LDL and total cholesterol (underlining the compliance of the volunteers to the dietary scheme) and the increase in SCFA [25], we show additional interesting evidence emerging from the same clinical trial. In fact, the beta-glucans dietary treatment was effective in reducing pCS blood levels in the study population, although the overall protein intake remained constant after the treatment, being the fiber content the only food component that significantly changed during the diet intervention. The slight induction in the glucose levels that we observed, although remaining in the normality range, could likely be related to this increased fiber intake during the intervention. Differently from pCS, IS did not change after the treatment. pCS and IS are produced respectively by phenylalanine/tyrosine and tryptophan degradation by the gut microbiota and are normally excreted through the urine. In the context of CKD, where the excretory function declines, they are emerging as the novel “uremic toxins” since they accumulate in blood in a proportion several-fold higher in comparison to healthy people [30,33]. Although directly correlated, as predictable from their common biosynthetic pathway, pCS and IS followed a different trend in our study. This apparent contradiction finds confirmation in some pieces of evidence in the literature, suggesting that pCS could be more susceptible than IS to intervention with food supplements [34,35]. The reasons for the differential modulation of pCS and IS by our dietary intervention are unknown and are worthy to be furtherly elucidated by future studies.

We previously demonstrated that the beta-glucans dietary treatment was able to induce a modulation of the gut microbiota taxonomic composition and metabolism, leading to an increase of SCFA levels [25]. This evidence, joined to the observed decrease of the circulating uremic toxin pCS, suggests—for the first time on a human study, according to our knowledge—the ability of beta-glucans to promote a saccharolytic shift in microbial metabolism.

Endothelial dysfunction is associated with several diseases, such as chronic heart failure and diabetes mellitus [32,36], and evidence demonstrated the vascular benefits of some nutrients in patients suffering from cardiovascular disease [37]. Notably, our study also evidences a positive effect of beta-glucans supplementation on the endothelial function in healthy subjects. Indeed, a recent interventional study with supplementation of 6 g of beta-glucans contained in a piece of oat bread failed to demonstrate a significant effect on FMD in hypercholesterolemic patients [27], although it increased serum NO levels [28]. In another study, the efficacy of whole oats and vitamin E to prevent endothelial dysfunction induced by a high-fat meal was demonstrated on healthy subjects, through brachial artery peak flow, but also in this case no difference in FMD was detected [26]. Differently from the aforementioned studies carried out in patients at higher cardiovascular risk, in this study we report a significant increase in FMD following the dietary treatment with the beta-glucans pasta. It is worth evidencing that it has been suggested that alterations of FMD could have a major predictive value in patients at low risk of cardiovascular events [38].

Interestingly, the inverse correlation we found between total cholesterol and FMD suggests a hypothetical mechanism of beta-glucans-induced amelioration of FMD through cholesterol reduction, even if our study does not allow us to confirm this hypothesis, neither we found significant correlations between changes of FMD, uremic toxins and cholesterol.

Recently, a link between pCS blood concentration and cardiovascular risk has been underlined, especially in the context of the renal failure [39–41]. In this pathology, microbial-derived proteolytic catabolites such as pCS and IS are not efficiently excreted by the kidney and accumulate in the blood, accelerating the disease progression and promoting inflammation, oxidative stress and cardiovascular complications [15]. In a recent paper, in particular, the independent association between pCS and IS with structural and functional markers of CVD was assessed, even if, as in the present study, no correlation was found between these uremic toxins and FMD [42]. It is of interest that the total serum concentrations of pCS might be a better predictor of CKD progression than IS [43] and that the association between plasma pCS levels and the risk of CVD is not limited to the CKD population but can also be found in patients with no renal diseases [44]. Finally, a recent meta-analysis indicated that elevated levels of pCS and IS are associated with increased mortality in patients with CKD, and pCS, but not IS, is associated with an increased risk of cardiovascular events [45].

We are aware of the limitations of the present study, in particular the small sample size, the short duration of the study and the lack of a parallel control group. The main consequence is that here we are just able to report the observed effects on metabolic microbial modulation and on endothelial function following beta-glucans ingestion, but not to formulate a mechanistic explanation of them, especially about the amelioration of FMD. In addition to the latter, the analysis of triglyceride rich lipoproteins (TGRLs) should have allowed us to investigate at molecular level the effects of beta-glucans on endothelial function [46,47].

Anyway, taking into consideration that the present research is a pilot study aimed to explore new healthy properties of barley beta-glucans, we can conclude that a balanced diet including a daily supply of beta-glucans, administrated for two months in healthy volunteers, is associated with a saccharolytic shift in the gut microbiota metabolism, evidenced by a reduction of pCS toxin blood levels and an increase of SCFA production at colonic site. Moreover, beyond reducing LDL and total cholesterol, beta-glucans treatment is associated with an amelioration of the endothelial function. The finding of this study could give support, if demonstrated in a larger scale and in a CKD context, to a recent and interesting branch of research [16–18,34,35] focusing on nutritional strategies to reduce uremic toxins, with the future aim to slow down CKD progression and reduce cardiovascular complications in end-stage renal disease.

## Supporting Information

S1 Fig Trend checklist. TREND checklist for non-randomized studies.
(PDF)

S2 Fig Fold changes of main parameters. Graphical representation of fold changes of total, LDL and HDL cholesterol, pCS, IS, FMD. Data are represented as mean ± SEM.
(TIF)

S1 File Related paper on beta-glucans effect on gut microbiota and metabolome. Effect of Whole-Grain Barley on the Human Fecal Microbiota and Metabolome. De Angelis M, Montemurno E, Vannini L, Cosola C, Cavallo N, Gozzi G et al. Appl Environ Microbiol. 2015; 81(22): 7945–7956.
(PDF)

S2 File Study protocol. Original study protocol as approved by the local Ethics Committee (document in Italian).
(DOCX)

S3 File Study protocol (English). English translation of the study protocol.
(DOCX)

S4 File Dietary advices. Mediterranean-based dietary advices–translation of the dietary advice distributed by the volunteers at the beginning of the study.
(DOCX)
