# Supplementary figures and images for: Beta-Glucans Supplementation Associates with Reduction in P-Cresyl Sulfate Levels and Improved Endothelial Vascular Reactivity in Healthy Individuals
Source: PLoS One. 2017 Jan 20;12(1):e0169635. doi: 10.1371/journal.pone.0169635 (PMC5249102; doi:10.1371/journal.pone.0169635)

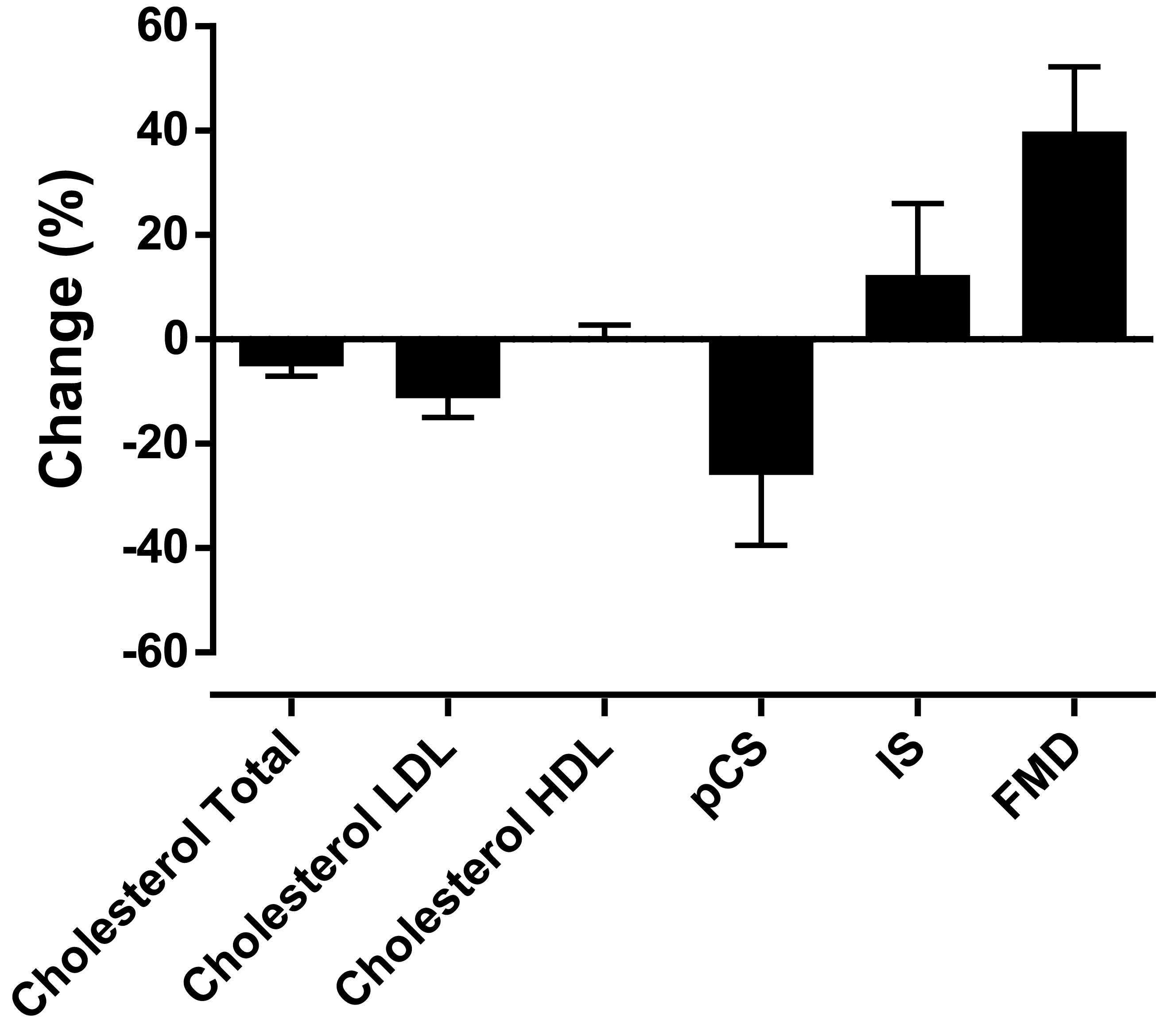

Supplement: S2 Fig — Graphical representation of fold changes of total, LDL and HDL cholesterol, pCS, IS, FMD. Data are represented as mean ± SEM. (TIF) [file pone.0169635.s002.tif]
