# Supplementary material for: Beta-Glucans Supplementation Associates with Reduction in P-Cresyl Sulfate Levels and Improved Endothelial Vascular Reactivity in Healthy Individuals
Source: PLoS One. 2017 Jan 20;12(1):e0169635. doi: 10.1371/journal.pone.0169635 (PMC5249102; doi:10.1371/journal.pone.0169635)
Supplement: S1 File — Effect of Whole-Grain Barley on the Human Fecal Microbiota and Metabolome. De Angelis M, Montemurno E, Vannini L, Cosola C, Cavallo N, Gozzi G et al. Appl Environ Microbiol. 2015; 81(22): 7945–7956. (PDF) [file pone.0169635.s003.pdf]

AEM Journals.ASM.org

# Effect of Whole-Grain Barley on the Human Fecal Microbiota and Metabolome

**Maria De Angelis,[a] Eustacchio Montemurno,[b] Lucia Vannini,[c,d] Carmela Cosola,[b] Noemi Cavallo,[a] Giorgia Gozzi,[c] Valentina Maranzano,[b] Raffaella Di Cagno,[a] Marco Gobbetti,[a] Loreto Gesualdo[b]**

Department of Soil, Plant and Food Sciences, University of Bari Aldo Moro, Bari, Italy[a]; Department of Emergency and Organ Transplantation, Nephrology Unit, University of Bari Aldo Moro, Bari, Italy[b]; Department of Agricultural and Food Sciences, University of Bologna, Bologna, Italy[c]; Interdepartmental Centre for Industrial Agri-Food Research, University of Bologna, Cesena, Italy[d]

**In this study, we compared the fecal microbiota and metabolomes of 26 healthy subjects before (HS) and after (HSB) 2 months of diet intervention based on the administration of durum wheat flour and whole-grain barley pasta containing the minimum recommended daily intake (3 g) of barley β-glucans. Metabolically active bacteria were analyzed through pyrosequencing of the 16S rRNA gene and community-level catabolic profiles. Pyrosequencing data showed that levels of *Clostridiaceae* (*Clostridium orbiscindens* and *Clostridium* sp.), *Roseburia hominis*, and *Ruminococcus* sp. increased, while levels of other *Firmicutes* and *Fusobacteria* decreased, from the HSB samples to the HS fecal samples. Community-level catabolic profiles were lower in HSB samples. Compared to the results for HS samples, cultivable lactobacilli increased in HSB fecal samples, while the numbers of *Enterobacteriaceae*, total coliforms, and *Bacteroides*, *Porphyromonas*, *Prevotella*, *Pseudomonas*, *Alcaligenes*, and *Aeromonas* bacteria decreased. Metabolome analyses were performed using an amino acid analyzer and gas chromatography-mass spectrometry solid-phase microextraction. A marked increase in short-chain fatty acids (SCFA), such as 2-methyl-propanoic, acetic, butyric, and propionic acids, was found in HSB samples with respect to the HS fecal samples. Durum wheat flour and whole-grain barley pasta containing 3% barley β-glucans appeared to be effective in modulating the composition and metabolic pathways of the intestinal microbiota, leading to an increased level of SCFA in the HSB samples.**

Whole-grain barley and oats and some dry-milled bran grain products were authorized for use according to the health claim made by the Food and Drug Administration that they "decrease the risk of coronary heart disease" (1, 2). Whole-grain barley and oat flours contain β-glucans, which are soluble dietary fibers. The European Food Safety Authority recognized that the "regular consumption of oat β-glucans can actively lower/reduce blood LDL-cholesterol and total cholesterol" (3). In addition to reducing cholesterol, the positive associations between the consumption of β-glucans and the reduction/prevention of cardiovascular diseases, as well as the reduction of glycemia, insulin resistance, and metabolic syndrome, have been well documented (4, 5). The minimum dose of 3 g/day of β-glucans is recommended to achieve positive effects on human health (1, 3, 6).

Several mechanisms were described to explain the hypocholesterolemic effect of β-glucans: (i) increased viscosity at the level of the small intestine and, consequently, slowed gastric emptying, digestion, and absorption of molecules, including glucose, dietary cholesterol, and bile acids (7, 8), (ii) decreased enterohepatic bile acids by binding at the intestinal level with subsequent increased use of cholesterol for bile acid synthesis (9, 10), (iii) reduced synthesis of hepatic cholesterol due to improved insulin sensitivity (10, 11), and (iv) inhibited hepatic synthesis of cholesterol by acetate and propionate, which are produced by colonic fermentation of β-glucans (12, 13). Saccharolytic and proteolytic fermentations are the major fermentation processes, which are carried out by metabolically active microbes at the colon level (14). Saccharolytic fermentation was associated with the synthesis of short-chain fatty acids (SCFA) (acetate, propionate, and butyrate), intermediate metabolites, such as succinate, acrylate, lactate, formate, and ethanol, and small final molecules (hydrogen, methane, and carbon dioxide) (15). Proteolytic fermentation was associated with the synthesis of SCFA (acetate, propionate, and butyrate) and branched-chain fatty acids (BCFA) (isobutyric, isovaleric, and 2-methylbutyric acids), free amino acids (FAA), and some potentially toxic metabolites (phenols, indoles, ammonia, and amines) (14, 16, 17). The type of colonic fermentation mainly depends on the type of microbiota and fermentable substrate availability (17). Based on the key role in human health, the intestinal microbiota was acknowledged as a metabolic organ (18). Most current research on novel functional foods is focused on the selection and characterization of prebiotics (e.g., inulin, fructooligosaccharides and galactooligosaccharides), which are not digested by human gastrointestinal enzymes but selectively stimulate the growth and/or activity of generally regarded as safe (GRAS) bacteria that may improve host health (19). Indeed, a prebiotic effect of β-glucans on the intestinal microbiota was also hypothesized (5). In particular, the positive effects of β-glucans on the growth of beneficial intestinal lactobacilli and bifidobacteria were shown in *in vitro* studies (20, 21) and animal experiments

**Received** 4 August 2015 **Accepted** 31 August 2015

**Accepted manuscript posted online** 18 September 2015

**Citation** De Angelis M, Montemurno E, Vannini L, Cosola C, Cavallo N, Gozzi G, Maranzano V, Di Cagno R, Gobbetti M, Gesualdo L. 2015. Effect of whole-grain barley on the human fecal microbiota and metabolome. Appl Environ Microbiol 81:7945–7956. doi:10.1128/AEM.02507-15.

**Editor:** J. Björkroth

Address correspondence to Marco Gobbetti, marco.gobbetti@uniba.it.

Supplemental material for this article may be found at http://dx.doi.org/10.1128/AEM.02507-15.

Copyright © 2015, American Society for Microbiology. All Rights Reserved.



Downloaded from http://aem.asm.org/ on October 22, 2015 by ALBERT EINSTEIN COLL OF MED

TABLE 1 Basic characteristics of volunteers[a]

| Characteristic | HS | HSB |
|---|---|---|
| Age (yr) | 39 ± 9 | 39 ± 9 |
| Male (%) | 46 | 46 |
| Body mass index (kg/m$^2$) | 22.6 ± 3 A[b] | 22.6 ± 3 A |
| Total cholesterol (mg/dl) | 183.8 ± 30.2 B | 173.25 ± 27.4 C |
| LDL cholesterol (mg/dl) | 107.4 ± 25.2 B | 93.25 ± 24.5 C |

[a] Each healthy subject was analyzed before (HS) and after (HSB) 2 months of diet intervention with 100 g/day of durum wheat and whole-grain barley pasta containing 3% (wt/wt) β-glucans. Values are means ± standard deviations.
[b] Values within a row followed by different letters are significantly different ($P < 0.05$).

(22, 23). Nevertheless, other studies that also used animal models did not show significant effects of β-glucans on lactobacilli and/or bifidobacteria (24, 25). Unfortunately, human clinical challenges that have dealt with the prebiotic effect of β-glucans are rather scarce. In one study, barley β-glucans increased the cell density of colonic bifidobacteria in older healthy subjects (26). A pilot study with polypectomized patients showed no significant effect of β-glucans on the fecal microbiota and concentration of SCFA (27). The evidence that β-glucans positively influence the human intestinal microbiota is still insufficient for interpretation or difficult to interpret, and additional studies are needed to fill this gap (5).

This study compared the fecal microbiota and metabolomes of healthy subjects before (HS) and after (HSB) 2 months of daily administration of durum wheat flour and whole-grain barley pasta containing the minimum recommended intake (3 g) of β-glucans. The fecal microbiota was characterized through integrated approaches, which were based on culture-independent and -dependent methods.

## MATERIALS AND METHODS

**Subjects.** The study was carried out in accordance with the Helsinki Declaration (IV adaptation) and European Guidelines for Good Clinical Practice. The protocol of the study was approved by the institutional review board of the Azienda Ospedaliero-Universitaria Consorziale Policlinico of Bari, Italy (authorization no. 1570/2014). Written consents were obtained from all volunteers. One group of Caucasian HS (15 female and 11 male), aged between 28 and 57 years, were enrolled in the study (Table 1). Exclusion criteria were history of gastrointestinal disease, diabetes, cardiovascular diseases, hyperlipidemia, and consumption of alcohol. Volunteers were not treated with antibiotics and/or functional foods (probiotics and/or prebiotics) for ≥3 months before recruitment and sampling.

**Feeding regime.** Pasta Granoro Cuore Mio (Granoro s.r.l., Corato, Bari, Italy) was used in this study. Pasta was made by using a mixture of durum wheat flour (75%) and whole-grain barley flour (25%). The gross composition of pasta Granoro Cuore Mio was as follows: moisture, 11%; protein, 11% of dry matter; carbohydrate, 69% of dry matter; fat, 2% of dry matter; total fibers and β-glucans, 7% and 3% of dry matter, respectively. Before the administration of 100 g of pasta Granoro Cuore Mio containing 3 g of barley β-glucans (Granoro s.r.l.), each volunteer was instructed to follow the usual diet, including 100 g of pasta every day for 2 months. Before and after the dietary treatment, a food frequency questionnaire and a 24-h recall questionnaire were administered to each volunteer by a dietitian. These two tools allowed extrapolation of the weekly cumulative frequency of food and the component intake data, respectively (Tables 2 and 3), by using official Italian food composition databases (Istituto Naxionale di Ricerca per gli Alimenti e la Nutrixione [INRAN] [http://nut.entecra.it/646/tabelle_di_composizione_degli_alimenti.html] and Istituto Europeo di Oncologia [IEO]). All volunteers confirmed that no remarkable changes occurred in their meals and medication during the 2 months of treatment. Additionally, a questionnaire aimed at evaluating intestinal effects (bloating, meteorism, constipation, satiety, diarrhea, reflux, and flatulence) was administered (26). Gastrointestinal symptoms were graded from 0 (no symptoms) to 3 (severe symptoms) (26). Before the treatment with durum wheat and whole-grain barley pasta, cholesterolemia, body mass index, and fecal microbiota and metabolome composition were determined.

**Determination of blood cholesterol.** The level of total cholesterol was measured on the Siemens Dimension RxL Max by using the Siemens enzymatic methods (Siemens Medical Solution Diagnostics, Tarrytown, NY). Low-density lipoprotein (LDL) and high-density lipoprotein (HDL) cholesterol levels were estimated by using the Friedewald equation (28) for values of <300 mg/dl.

TABLE 2 Weekly diet of volunteers[a]

| Component | Weekly cumulative frequency (%) of food eaten: HS | HSB |
|---|---|---|
| Milk | 50 A[b] | 50 A |
| Pasta | 100 A | 0 B |
| Pasta β-glucan | 0 A | 100 B |
| Bread | 97 A | 100 A |
| Meats | 85 A | 84 A |
| Cured meats | 73 A | 68 A |
| Fish | 77 A | 81 A |
| Dairy products | 81 A | 77 A |
| Eggs | 89 A | 84 A |
| Legumes | 77 A | 76 A |
| Leafy vegetables | 100 A | 100 A |
| Fruits | 93 A | 96 A |
| Yogurt | 8 A | 8 A |
| Sweets | 59 A | 57 A |

[a] Each healthy subject was analyzed before (HS) and after (HSB) 2 months of diet intervention with 100 g/day of durum wheat and whole-grain barley pasta containing 3% (wt/wt) β-glucans.
[b] Values within a row followed by different letters are significantly different ($P < 0.05$).

TABLE 3 Component intake by volunteers[a]

| Component | HS | HSB |
|---|---|---|
| Water (g) | 717.0 ± 274.6 A[b] | 853.4 ± 512.7 A |
| Protein (g) | 69.9 ± 29.5 A | 77.6 ± 41.7 A |
| Lipid (g) | 58.3 ± 31.3 A | 68.7 ± 42.2 A |
| Carbohydrate (g) | 199.8 ± 73.9 A | 237.4 ± 101.6 A |
| Starch (g) | 130.1 ± 54.9 A | 151.1 ± 70.3 A |
| Soluble sugars (g) | 58.7 ± 27.9 A | 73.4 ± 37.6 A |
| Alcohol (g) | 7.9 ± 13.3 A | 6.5 ± 12.1 A |
| Total fiber (g) | 13.4 ± 8.2 A | 22.0 ± 11.7 B |
| Energy (kcal) | 1,613.1 ± 609.6 A | 1,894.3 ± 908.9 A |
| Sodium (mg) | 1,233.3 ± 886.5 A | 1,498.5 ± 1,180.4 A |
| Potassium (mg) | 2,091.7 ± 888.6 A | 2,408.5 ± 1,433.0 A |
| Iron (mg) | 11.7 ± 20.7 A | 8.3 ± 6.1 A |
| Calcium (mg) | 446.2 ± 274.6 A | 533.0 ± 339.8 A |
| Phosphorus (mg) | 986.1 ± 705.8 A | 940.6 ± 625.7 A |
| Thiamine (mg) | 0.7 ± 0.3 A | 0.9 ± 0.8 A |
| Riboflavin (mg) | 0.9 ± 0.4 A | 1.0 ± 0.8 A |
| Niacin (mg) | 11.5 ± 6.3 A | 12.78.5 A |
| Vitamin C (mg) | 64.6 ± 39.7 A | 179.3 ± 340.3 A |
| Vitamin E (mg) | 5.0 ± 8.9 A | 5.8 ± 12.1 A |

[a] Each healthy subject was analyzed before (HS) and after (HSB) 2 months of diet intervention with 100 g/day of durum wheat and whole-grain barley pasta containing 3% (wt/wt) β-glucans.
[b] Values within a row followed by different letters are significantly different ($P < 0.05$).

Downloaded from http://aem.asm.org/ on October 22, 2015 by ALBERT EINSTEIN COLL OF MED

**Collection of fecal samples.** Each volunteer had fasted overnight, and a preprandial fecal sample was collected in the morning. Fecal samples were collected on three different days of the same week. After collection, samples were immediately mixed with RNA*later* (Sigma-Aldrich, St. Louis, MO, USA) (ca. 5 g, 1:2 [wt/vol]) or Amies transport medium (Oxoid Ltd., Basingstoke, Hampshire, England) (ca. 15 g, 1:1 [wt/wt]), under anaerobic conditions (AnaeroGen, Oxoid Ltd., Basingstoke, Hampshire, England). Fecal samples suspended in RNA*later* were stored at −80°C for further RNA and metabolomic analyses. Samples diluted with Amies transport medium were immediately subjected to plate counts and analysis by the Biolog system.

**RNA extraction from fecal samples.** An aliquot of ca. 200 mg of fecal sample diluted in RNA*later* was used for RNA extraction with the stool total RNA purification kit (Norgen Biotek Corp., Ontario, Canada). The quality and concentration of RNA extracts were determined using 1% agarose–0.5× Tris buffer-EDTA (TBE) gels and spectrophotometric measurements at 260, 280, and 230 nm through the NanoDrop ND-1000 spectrophotometer. Total RNA extracted (ca. 2.5 μg) was transcribed to cDNA using random examers and the Tetro cDNA synthesis kit (Bioline USA, Inc., Taunton, MA, USA), according to the manufacturer's instructions (29).

**bTEFAP and data analyses.** For each volunteer, the three cDNA samples were pooled and used for bacterial tag-encoded FLX amplicon pyrosequencing (bTEFAP) analysis. Pooled samples of cDNA were analyzed for each subject. bTEFAP was performed by Research and Testing Laboratories (Lubbock, TX), according to standard laboratory procedures and using the 454 FLX sequencer (454 Life Sciences, Branford, CT, USA). The primers were forward 28F (GAGTTTGATCNTGGCTCAG) and reverse 519R (GTNTTACNGCGGCKGCTG), based upon the V1 to V3 region (*Escherichia coli* positions 27 to 519) of the 16S rRNA gene (30). The bTEFAP procedures were performed based upon Research and Testing Laboratories (RTL) protocols (Research and Testing Laboratories, Lubbock, TX). Raw sequence data were screened, trimmed, and filtered with default settings, using the QIIME pipeline version 1.4.0 (http://qiime.org/). Chimeras were excluded by using B2C2 (Research and Testing) (31). Sequences of <250 bp were removed. The average length of the sequences was 484 bp. The sequences are available on the Research and Testing Laboratories website. The FASTA sequences for each sample, without chimeras, were evaluated using BLASTn against the database derived from GenBank (http://ncbi.nlm.nih.gov) (32).

**Bioinformatics and data analysis.** The sequences were first grouped into operational taxonomic unit (OTU) clusters with 97% identity (3% divergence), using USEARCH (33). To determine the identities of bacteria, sequences were first queried using a distributed BLASTn.NET algorithm (33) against the database of high-quality 16S bacterial sequences that were derived from the NCBI. Database sequences were characterized as high quality based on criteria originally described by Ribosomal Database Project (RDP) version 10.28 (34).

Alpha diversity (determined by rarefaction, Good's coverage, Chao1 richness, and Shannon diversity indices) and beta diversity measures were calculated and plotted using QIIME. Diversity was examined from two perspectives. First, overall richness (i.e., number of distinct organisms present within the microbiome) (alpha diversity) was expressed as the number of OTUs and was quantified using the Chao1 richness estimator: $S_{chao1} = S_{obs} + (n1\ [n1 - 1]/2[n2 + 1])$, where n1 is the number of singletons (species captured once) and n2 is the number of doubletons (species captured twice).

Second, overall diversity (which is determined by both richness and evenness and the distribution of abundance among distinct taxa) was expressed using the Shannon diversity index. The Shannon diversity index ($H'$) is calculated using the equation $H' = -\sum_{i=1}^{R} P_i \times \ln(P_i)$, where $R$ is richness and $p_i$ is the relative abundance of the $i$th OTU.

Measures of diversity were screened for group differences by using analysis of variance (ANOVA). Multivariate differences among groups were evaluated with permutational multivariate analysis of variance using the distance matrices function in Adonis (35). For Adonis, distances between samples were calculated using unweighted or weighted UniFrac, and then an ANOVA-like simulation was conducted to test for group differences. In addition, multivariate differences were assessed using the analysis of similarities function in anosim (35). Sequence data were processed using a Research and Testing pipeline that is described at http://www.researchandtesting.com/docs/Data_Analysis_Methodology.pdf. Spearman correlations between OTU and metabolite concentration were computed. All analyses were conducted in R, using the vegan (35), labdsv (36), DESeq2 (37), and phyloseq (38) packages.

**Community-level catabolic profiles.** Carbon source utilization patterns of the fecal microbiota were assessed using Biolog 96-well Eco microplates (Biolog, Inc., Hayward, CA, USA) (39). The microplates contained 31 different carbon sources (carbohydrates, carboxylic acids, polymers, amino acids, amines, and miscellaneous substrates) in triplicate. Five grams of feces diluted with Amies transport medium (1:1) was homogenized in a filter bag with 45 ml of 0.9% sterile sodium chloride (wt/vol) solution (Classic Blender) to remove fecal solid particulate. The homogenized feces were centrifuged at 11,000 rpm for 15 min at 4°C. The pellet was washed with 50 mM Tris-HCl (pH 7.0) and then with sterile sodium chloride solution and centrifuged at 11,000 rpm for 15 min at 4°C. The cellular suspension was diluted (1:10) into the sterile sodium chloride solution and subsequently centrifuged at 2,000 rpm for 2 min at 4°C. The cellular suspension was diluted (1:20) into sterile chloride solution and dispensed (150 μl) into each of the 96 wells of the Biolog Eco microplates. The microplates were incubated at 37°C in the dark under anaerobic conditions and with slow stirring. The color development was measured at 590 nm every 24 h with a microplate reader (MicroStation; Biolog, Inc.). Three indices were determined (40). Shannon's diversity ($H'$), indicating the substrate utilization pattern, was calculated as follows: $H' = -\Sigma\, p_i \times \ln(p_i)$, where $p_i$ is the ratio of the activity of a particular substrate to the sums of the activities of all substrates at 120 h. Substrate richness ($S$), measuring the number of different substrates used, was calculated as the number of wells with a corrected absorbance of >0.25. Substrate evenness ($E$) was defined as the equitability of activities across all utilized substrates: $E = H'/\log S$.

**Enumeration of cultivable bacteria.** Diluted fecal samples (20 g) were mixed with 80 ml of sterilized physiological solution and homogenized. Counts of viable bacterial cells were carried out as described by De Angelis et al. (41). The following selective media were used: Wilkins-Chalgren anaerobe agar (for total anaerobes), deMan, Rogosa, and Sharpe (MRS) agar (for *Enterococcus* and *Lactobacillus*), Slanetz and Bartley (for *Enterococcus*), Rogosa agar plus 1.32 ml/liter glacial acetic acid (for *Lactobacillus*), M17 (for *Lactococcus* and *Streptococcus*), Baird Parker (for *Staphylococcus*), Wilkins-Chalgren anaerobe agar plus Gram-negative (GN) selective supplements and defibrinated sheep blood (for *Bacteroides*, *Porphyromonas*, and *Prevotella*), MacConkey agar no. 2 (for *Enterobacteriaceae*), ChromoCult (for total coliforms) (Merck, Darmstadt, Germany), glutamate starch *Pseudomonas* (GSP) agar (Sigma-Aldrich, St. Louis, MO, USA) plus penicillin G (60 g/liter) (for *Pseudomonas*, *Alcaligenes*, and *Aeromonas*), modified *Bifidobacterium* agar (for *Bifidobacterium*) (Becton Dickinson, Le Pont de Claix, SA, France), and Hoyle medium (for *Corynebacterium*). Except for the modified *Bifidobacterium* agar, ChromoCult, and GSP agar, all media were purchased from Oxoid Ltd. (Basingstoke, Hampshire, England).

**Fecal concentration of free amino acids.** FAA of fecal samples were analyzed through the Biochrom 30 series amino acid analyzer (Biochrom Ltd., Cambridge Science Park, England) with a sodium cation-exchange column (20 cm long by 0.46 cm inner diameter). A mixture of amino acids at known concentrations (Sigma Chemical Co., Milan, Italy) was added to cysteic acid, methionine sulfoxide, methionine sulfone, tryptophan, ornithine, glutamic acid, and γ-aminobutyric acid and used as the standard. Proteins and peptides in the fecal samples were precipitated by adding 5% (vol/vol) cold solid sulfosalicylic acid, holding the samples at 4°C for 1 h, and centrifuging at 15,000 × $g$ for 15 min. The supernatant was filtered

Downloaded from http://aem.asm.org/ on October 22, 2015 by ALBERT EINSTEIN COLL OF MED

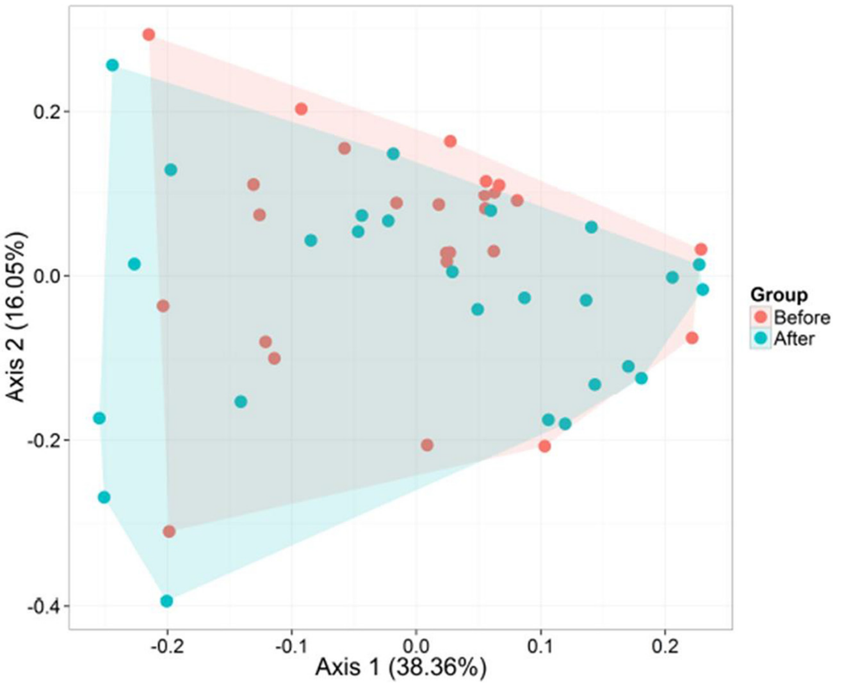


**FIG 1** Principal coordinate analysis (PCA) of metabolically active bacteria. PCA was based on weighted UniFrac analysis of all 16S rRNA gene sequences found in fecal samples of healthy subjects before (HS) and after (HSB) 2 months of diet intervention with durum wheat flour and whole-grain barley pasta.

through a 0.22-μm-pore-size filter and diluted when necessary with sodium citrate loading buffer (0.2 M [pH 2.2]). Amino acids were postcolumn derivatized with ninhydrin reagent and detected by absorbance at 440 nm (for proline and hydroxyproline) or 570 nm (for all other amino acids).

**Gas chromatography-mass spectrometry solid-phase microextraction analysis of fecal volatile compounds.** After preconditioning, according to the manufacturer's instructions, a polydimethylsiloxane/divinylbenzene fiber (65 μm) and a manual solid-phase microextraction (SPME) holder (Supelco, Inc., Bellefonte, PA, USA) were used. Before headspace sampling, the fiber was exposed to a gas chromatography (GC) inlet for 1 h for thermal desorption at 250°C. Three grams of fecal sample was placed into 10-ml glass vials and added to 10 μl of 4-methyl-2-pentanol (final concentration, 33 mg/liter) as the internal standard. The samples were then equilibrated for 10 min at 40°C. SPME fiber was exposed to each sample for 40 min. Both the equilibration and absorption phases were carried out with stirring. The fiber was then inserted into the injection port of the gas chromatograph for 10 min of sample desorption. Gas chromatography-mass spectrometry (GC-MS) analyses were carried out with an Agilent 7890A gas chromatograph (Agilent Technologies, Palo Alto, CA) coupled to an Agilent 5975C mass selective detector operating in electron impact mode (ionization voltage, 70 eV). A Supelcowax 10-capillary column (length, 60 m; inside diameter, 0.32 mm; Supelco, Inc.) was used. The temperature program was 50°C for 1 min, followed by an increase at a rate of 4.5°C/min to 65°C, an increase at a rate of 10°C/min to 230°C, and then 230°C for 25 min. The injector, interface, and ion source temperatures were 250, 250, and 230°C, respectively. The mass-to-charge ratio interval was 30 to 350 Da at a rate of 2.9 scans per s. Injection was carried out in splitless mode, and helium (flow rate, 1 ml/min) was used as the carrier gas. Molecules were identified based on a comparison of their retention times with those of pure compounds (Sigma-Aldrich, Milan, Italy). Identities were confirmed by searching mass spectra in the available databases (NIST version 2005 and Wiley version 1996). Quantitative data for the identified compounds were obtained by interpolation of the relative areas versus the internal standard area (42). All GC-MS/SPME raw files were converted to netCDF format via ChemStation (Agilent Technologies, USA) and subsequently processed by the XCMS toolbox (https://xcmsonline.scripps.edu/). XCMS software allows automatic and simultaneous retention time alignment, matched filtration, peak detection, and peak matching. GC-MS/SPME data were organized into a matrix for subsequent statistical analysis.

**Statistical analysis.** Culture-dependent data were obtained at least in triplicate. The analysis of variance (ANOVA) was carried out on transformed data, followed by the separation of means with Tukey's honestly significant difference (HSD) test, using the statistical software Statistica 6.0 for Windows 1998 (StatSoft, Vigonza, Italy). In order to identify differences between HS and HSB samples, GC-MS/SPME data were analyzed by canonical discriminant analysis of principal coordinates (CAP) (41). The total variance obtained in the principal coordinates used to perform the CAP was <80% for all samples. Significance testing was carried out using 999 permutations. The correctly performed permutation test assigns ca. 90% of the samples. Moreover, models based on projection to latent structures (PLS) in its discriminant (DA) version were built based on the normalized concentration of the significant molecules that were identified (43). Metabolically active bacterial families/genera and fecal volatile compounds were analyzed by principal component analysis (PCA) using the statistical software Statistica 6.0 for Windows 1998 (StatSoft).

**Nucleotide sequence accession number.** The sequence data were submitted to the Sequence Read Archive database of the National Center for Biotechnology Information under accession no. PRJNA290897.

## RESULTS

**Diet and clinical evaluation.** In this study, the diet of the volunteers was monitored for 2 months before the administration of durum wheat flour and whole-grain barley pasta containing the minimum recommended intake (3 g) of β-glucans. This was done to ascertain that every volunteer consumed equivalent amounts of carbohydrates, total protein, and fat. The only statistically signif-

Downloaded from http://aem.asm.org/ on October 22, 2015 by ALBERT EINSTEIN COLL OF MED

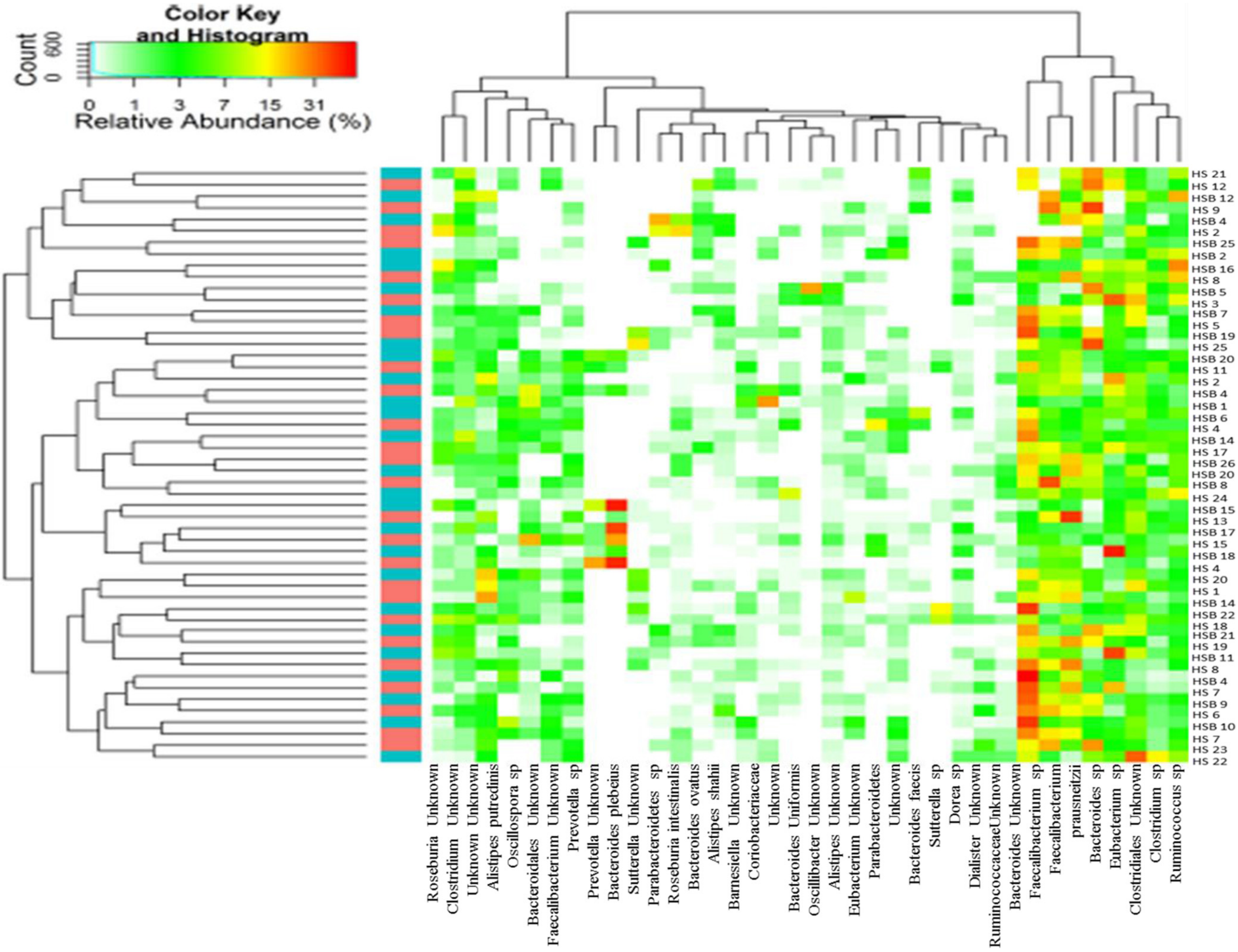


**FIG 2** Heatmap summarizing the relative abundances of the 35 most dominant species in RNA samples directly extracted from healthy subjects before (HS) and after (HSB) 2 months of diet intervention with durum wheat flour and whole-grain barley pasta. The color key defines the percentages of OTUs in the samples.

icant ($P < 0.05$) difference in the diet, before and after administration of durum wheat flour and whole-grain barley pasta, was the amount of total fiber (Table 3). Barley flour, including that with β-glucans, was well tolerated by all volunteers, and no statistically significant ($P > 0.05$) effects regarding bloating, abdominal pain score, flatulence, or frequency or consistency of evacuations of feces were observed (see Table S1 in the supplemental material). The observed reduction in non-HDL cholesterol confirmed the European Food Safety Authority healthy claim (3). The estimation of cholesterol amount was used as an internal control to validate the adherence of each volunteer to the diet and, consequently, the consistency of the study.

**Richness and diversity of the fecal microbiota based on 16S rRNA gene sequencing data analysis.** Total metabolically active bacteria from fecal samples of healthy subjects before (HS) and after (HSB) the administration of durum wheat flour and whole-grain barley pasta were analyzed by pyrosequencing of the 16S rRNA gene. After quality control, pyrosequencing analysis yielded 156,563 total bacterial read sequences, with a mean of 3,010 reads (range, 1,146 to 5,621 reads) per sample. The

**TABLE 4** Pyrosequencing data summary[a]

| Organism | Family or species | Avg % HS[b] | Avg % HSB[b] | P value HS/HSB |
|---|---|---|---|---|
| *Firmicutes* | *Clostridiaceae* | 5.26 | 8.08 | 0.019 |
| | *Eubacteriaceae* | 8.827 | 5.135 | 0.229 |
| | *Ruminococcaceae* | 31.55 | 23.37 | 0.023 |
| *Fusobacteria* | *Fusobacteriaceae* | 3.06 | 1.85 | 0.019 |
| *Firmicutes* | *Clostridium orbiscindens* | 0.22 | 0.68 | 0.045 |
| | *Clostridium* sp. | 2.95 | 4.69 | 0.048 |
| | *Roseburia hominis* | 0.01 | 0.12 | 0.047 |
| | *Faecalibacterium prausnitzii* | 12.27 | 6.08 | 0.004 |
| | *Faecalibacterium* sp. | 11.29 | 6.32 | 0.016 |
| | *Ruminococcus* sp. | 5.60 | 8.82 | 0.047 |
| | *Dialister invisus* | 0.52 | 0.13 | 0.034 |
| *Fusobacteria* | *Fusobacterium* sp. | 3.06 | 1.85 | 0.019 |

[a] Each healthy subject was analyzed before (HS) and after (HSB) 2 months of diet intervention with 100 g/day of durum wheat and whole-grain barley pasta containing 3% (wt/wt) β-glucans.
[b] Avg %, relative abundance of predominant bacterial taxa, showing significant ($P < 0.05$) differences between fecal samples for HS and HSB.

Downloaded from http://aem.asm.org/ on October 22, 2015 by ALBERT EINSTEIN COLL OF MED

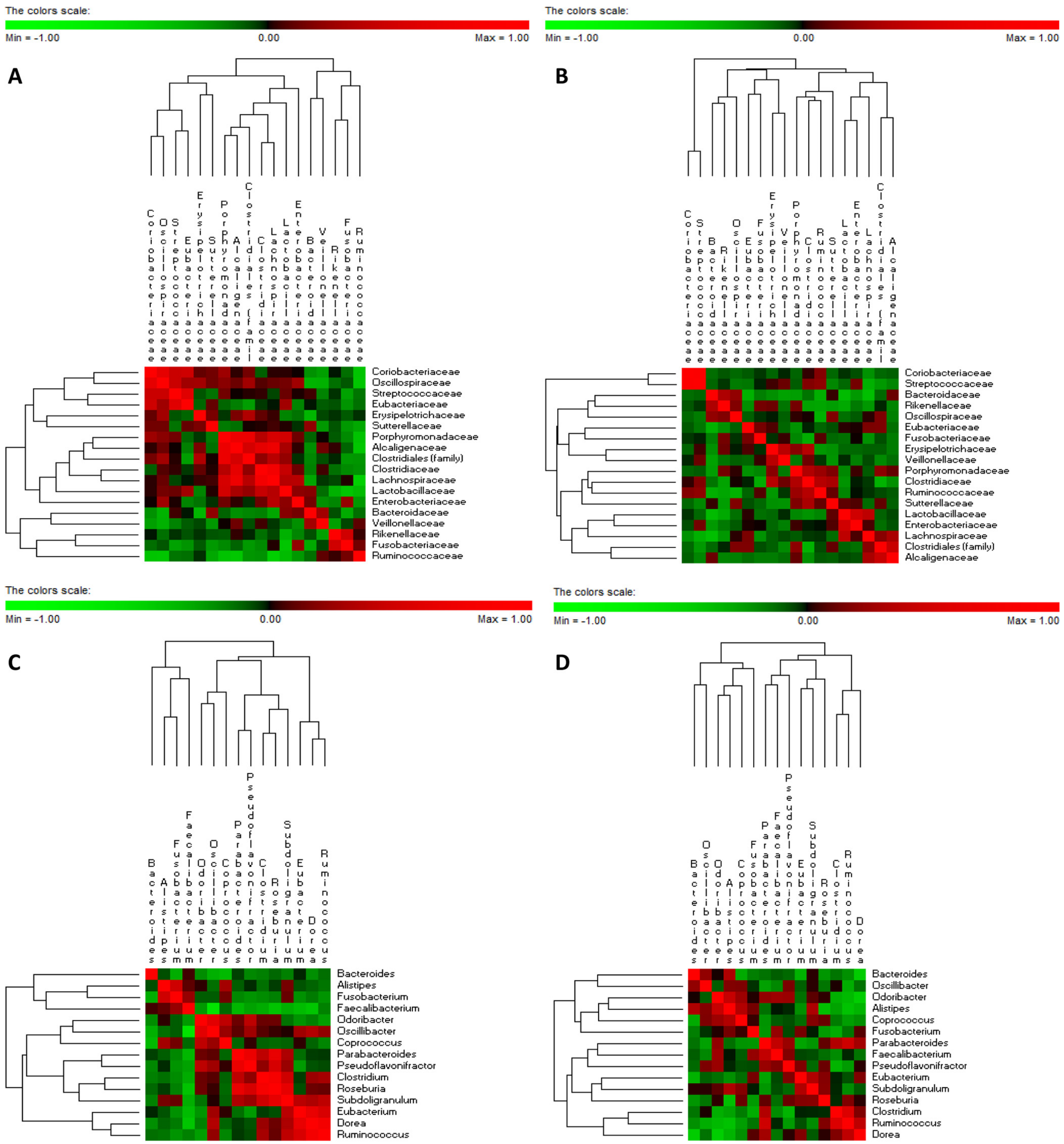


**FIG 3** Significant correlations between family-level (A and B) and genus-level (C and D) bacterial OTUs before (A and C) and after (B and D) 2 months of diet intervention with durum wheat flour and whole-grain barley pasta. The colors of the scale bar denote the nature of the correlation, with 1 indicating a perfectly positive correlation (red) and −1 indicating a perfectly negative correlation (green) between two microbial families or genera. Only significant correlations (FDR, <0.05) are shown.

metabolically active bacterial community was analyzed by rarefaction curves (see Fig. S1 in the supplemental material), estimated operational taxonomic units (OTUs), a richness estimator (Chao1), and the diversity index (Shannon). The Good's estimated sample coverage was ca. 97%. The mean numbers of estimated OTUs were 127.19 and 132 ($P = 0.605$) for HS and HSB samples, respectively. According to the OTU values, the mean values of Chao1 (152 versus 160.8, respectively) and Shannon index (3.27 versus 3.3, respectively) were not significantly different ($P = 0.466$ and 0.794 for Chao1 and Shannon



Downloaded from http://aem.asm.org/ on October 22, 2015 by ALBERT EINSTEIN COLL OF MED

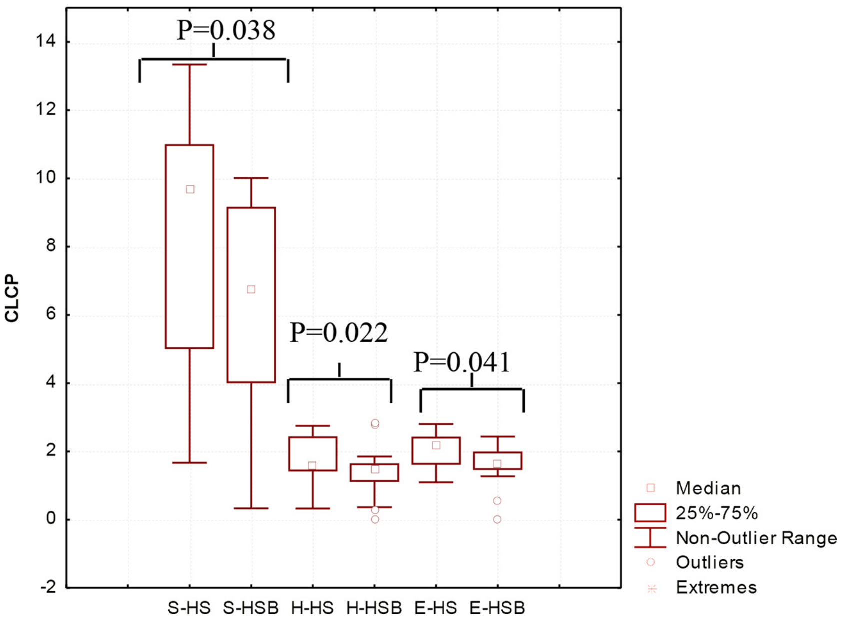


**FIG 4** Community-level catabolic profile (CLCP) indices (utilization pattern substrate [$H'$], substrate richness [$S$], and equitability [$E$]) of the fecal microbiota of healthy subjects before (HS) and after (HSB) 2 months of diet intervention with durum wheat flour and whole-grain barley pasta. The data are the results from three independent experiments ($n = 3$). The center line of each box represents the median, and the top and bottom of the box represent the 75th and 25th percentiles of the data, respectively. The top and bottom of the error bars represent the 95th and 5th percentiles of the data, respectively. The circles in each box plot extend to the outliers of the data.

index, respectively) between HS and HSB samples (see Fig. S2 in the supplemental material).

According to the alpha-diversity values, the three phylogeny-based beta-diversity measures did not show a clear separation between the microbiota compositions of HS and HSB samples in weighted and unweighted UniFrac distance principal coordinate analysis plots (Fig. 1; see also Fig. S3 in the supplemental material). Also, the Adonis statistical test indicated no significant ($P = 0.199$) differences between the microbial diversities of HS and HSB samples. However, anosim results, which were based on weighted UniFrac testing for multivariate differences, differed ($P = 0.04$; TestStat, 0.05) between HS and HSB samples. Overall, seven phyla (*Actinobacteria*, *Bacteroidetes*, *Firmicutes*, *Fusobacteria*, *Lentisphaerae*, *Proteobacteria*, and *Verrucomicrobia*) and one candidate division (TM7) were identified (see Fig. S4 in the supplemental material). However, *Firmicutes* and *Bacteroidetes* represented >85% of all 16S rRNA sequences. No significant ($P > 0.05$) differences were found for the relative abundances of the phyla between HS and HSB samples. The only exception was the phylum *Fusobacteria*, which was higher in HS than in HSB samples (average relative abundance, 3.06 versus 1.85%, respectively; $P = 0.019$).

According to alpha and beta diversity results and considering the 35 most dominant OTUs (representing 95% of the total microbiota) in all fecal samples, HS and HSB volunteers were variously distributed (Fig. 2). Few statistically significant ($P < 0.05$) differences were detected between the fecal microbiota of HS samples and that of HSB samples (Table 4). HSB samples showed the highest relative abundances of *Clostridiaceae* (*Clostridium orbiscindens* and *Clostridium* sp.), *Lachnospiraceae* (*Roseburia hominis*), and *Ruminococcus* sp. Abundances of other *Ruminococcaceae* (*Faecalibacterium prausnitzii* and *Faecalibacterium* sp.), *Dialister invisus*, and *Fusobacteriaceae* (*Fusobacterium* sp.) were the lowest in HSB samples.

**OTU correlation.** OTU correlations were investigated considering family-level (Fig. 3A and B) and genus-level (Fig. 3C and D) taxonomic assignments, with significant correlations considered at a false discovery rate (FDR) of <0.050. Several family-related positive correlations were always found (e.g., *Coriobacteriaceae* with *Streptococcaceae* and *Porphyromonadaceae* with *Clostridiaceae*). Other positive correlations found in HS samples (e.g., *Eubacteriaceae* with *Coriobacteriaceae*, *Oscillospiraceae*, and *Streptococcaceae*) were not confirmed in HSB samples. A similar trend was detected also at the genus level. The only exception was for *Bacteroides*, which showed an increased number of positive correlations in HSB samples compared to that in HS samples.

**Community-level catabolic profiles and cultivable bacteria.** The substrate utilization pattern ($H'$ index) and substrate richness ($S$ index) values were calculated (Fig. 4). The $H'$ and $S$ indices of the HSB samples were lower than those of the HS samples. The $E$ index, which measures the statistical significance (equitability) of the $H'$ and $S$ index values, confirmed the above-described significant ($P < 0.05$) differences.

Selective media were used to enumerate cultivable bacteria (Table 5). HSB samples had fewer total anaerobes than HS samples ($P = 0.05$). The median numbers of presumptive *Lactobacillus* in HS samples were lower than those in HSB samples. Other significant ($P < 0.05$) differences concerned the numbers of *Enterobacteriaceae*, total coliforms, and presumptive *Bacteroides*, *Porphyromonas*, *Prevotella*, *Pseudomonas*, *Alcaligenes*, and *Aeromonas* bacteria, which were the lowest in the HSB fecal samples.

**Fecal metabolome.** Fecal samples from HSB had lower ($P < 0.05$) levels of some FAA (Pro, Trp, Thr, and Arg) and metabolites from the catabolism of FAA (e.g., γ-aminobutyric acid)

Downloaded from http://aem.asm.org/ on October 22, 2015 by ALBERT EINSTEIN COLL OF MED

**TABLE 5** Cultivable fecal bacteria of the main microbial groups

| Microbial group | No. of cultivable cells (mean [range]) (log CFU/ml) in[a]: HS | HSB |
|---|---|---|
| Total anaerobes | 9.80 (9.33–9.93) A[b] | 8.42 (4.98–9.81) B |
| *Lactobacillus thermophilus* (42°C) | 6.61 (4.60–8.93) B | 7.53 (4.93–9.16) A |
| *Lactobacillus mesophilus* (25°C) | 6.61 (3.26–8.98) B | 7.38 (4.46–9.28) A |
| *Enterococcus* | 7.05 (5.36–8.55) A | 7.29 (3.90–9.19) A |
| *Lactococcus* and *Streptococcus* | 7.74 (5.36–8.55) A | 7.57 (4.25–9.26) A |
| *Staphylococcus* | 5.68 (2.84–8.11) A | 6.14 (4.38–8.08) A |
| *Bacteroides*, *Porphyromonas*, and *Prevotella* | 9.09 (3.00–9.92) A | 5.17 (2.52–6.44) B |
| *Enterobacteriaceae* | 7.07 (5.11–9.27) A | 6.11 (4.19–7.14) B |
| Total coliforms | 6.94 (1.91–8.93) A | 6.48 (1.00–5.32) B |
| *Pseudomonas*, *Alcaligenes*, and *Aeromonas* | 6.42 (1.97–7.95) A | 3.68 (1.00–4.81) B |
| *Bifidobacterium* | 7.16 (4.33–9.80) A | 6.90 (4.19–9.09) A |
| *Corynebacterium* | 4.93 (1.74–7.68) A | 5.35 (2.00–7.17) A |

[a] Each healthy subject was analyzed before (HS) and after (HSB) 2 months of diet intervention with 100 g/day of durum wheat and whole-grain barley pasta containing 3% (wt/wt) β-glucans.
[b] Values within a row followed by different letters are significantly different ($P < 0.05$).

than samples from HS (see Fig. S5 in the supplemental material). The levels of some volatile organic compounds (VOC), which were detected in fecal samples of HS, markedly differed in HSB samples (Fig. 5A; see also Fig. S6 in the supplemental material). Compared to HS samples, fecal samples of HSB showed an increased content of several short-chain fatty acids (SCFA) (2-methyl-propanoic acid, acetic acid, butanoic acid, and propanoic acid) (Fig. 5B). Pentanoic acid was the only SCFA found at the highest level in HS samples. The other statistically significant differences for VOC regarded the levels of phenylethyl alcohol, benzaldehyde, indole, 2,3-butanedione, 6-methyl-5-hepten-2-one, and acetophenone, which were the highest in HSB samples. FAA and GC-MS/SPME data were also analyzed using the principal component analysis (PCA). The discrimination of fecal samples between HS and HSB was evident (see Fig. S7 in the supplemental material).

**OTU-metabolite correlations.** Correlations between metabolically active bacterial families/genera and metabolomic data (FAA and VOC) were found (Fig. 6). *Coriobacteriaceae*, *Streptococcaceae*, *Faecalibacterium*, *Ruminococcaceae*, and *Ruminococcus* were positively correlated with hexanoic and propanoic acids. Other positive correlations were found for *Ruminococcaceae* and Tpr and γ-aminobutyric acid and for *Ruminococcus* and Tpr, γ-aminobutyric acid, acetic acid, and butanoic acid. Hexanoic acid was also positively correlated with *Parabacteroides*, *Clostridiaceae*, and *Clostridium*. *Bacteroidaceae*, *Bacteroides*, and *Alcaligenaceae* were positively correlated with Tpr, acetic acid, butanoic acid, propanoic acid, $NH_3$, indole, Arg, acetophenone, and benzaldehyde. *Roseburia* and *Lachnospiraceae* showed various positive correlations, including with Thr, γ-aminobutyric acid, acetic acid, butanoic acid, $NH_3$, indole, propanoic acid, phenyl ethyl alcohol, and 2,3-butanedione.

## DISCUSSION

This is one of the few studies showing the effects of the consumption of durum wheat flour and whole-grain barley pasta on the human fecal microbiota and metabolome, using a metaomics approach. As shown through pyrosequencing analysis, the intervention with durum flour wheat and whole-grain barley pasta, resulting in the ingestion of 3 g/day of β-glucans, did not affect the values of alpha and beta diversity. Previously, high-throughput sequencing techniques revealed that alpha diversity decreased with dietary supplementation of β-glucans from sea cucumber (*Apostichopus japonicus*) (44) and mirror carp (*Cyprinus carpio*) (45). The phylogenetic composition of the analyzed samples confirms that the *Firmicutes* and *Bacteroidetes* phyla constitute the most abundant bacterial OTUs of human intestinal microbiota. The composition of the main bacterial phyla (*Firmicutes* and *Bacteroidetes*) within the enrolled volunteers was variously affected, without a unique statistically significant trend. According to the 16S rRNA gene-based high-throughput sequencing, a wide variation was found between individuals (46, 47). Levels of *Clostridi-*

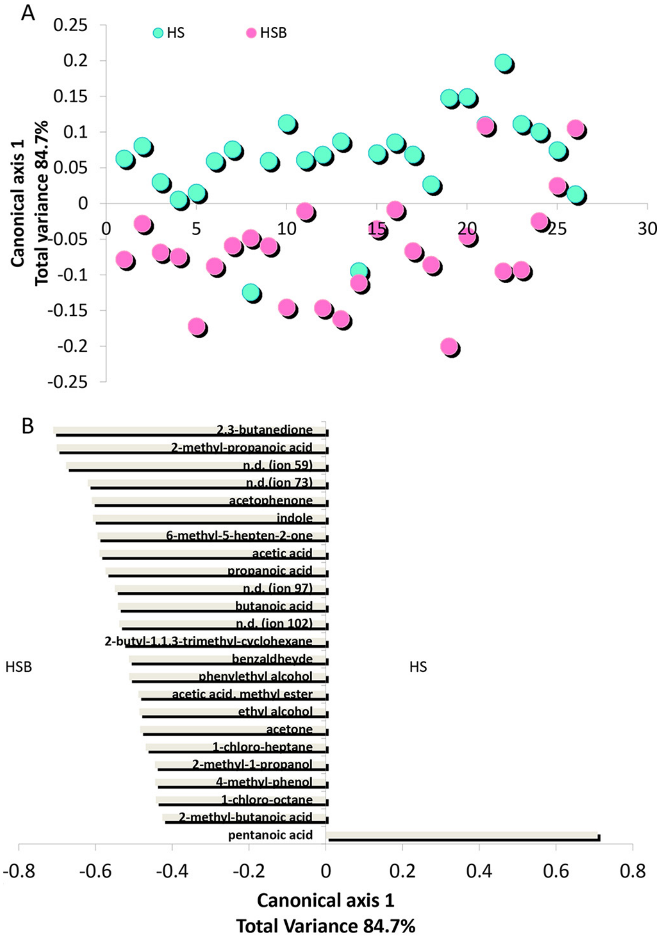


**FIG 5** Score (A) and loading coefficient (B) plots of canonical discriminant analysis of principal coordinates (CAP) of volatile organic metabolites in the feces of healthy subjects before (HS) and after (HSB) diet intervention with durum wheat flour and whole-grain barley pasta. Compounds significantly associated with the feces of HSB are shown on the negative axis; those associated with HS samples are shown on the positive axis. The data are the means of the results from three independent experiments ($n = 3$). n.d., not determined.

Downloaded from http://aem.asm.org/ on October 22, 2015 by ALBERT EINSTEIN COLL OF MED

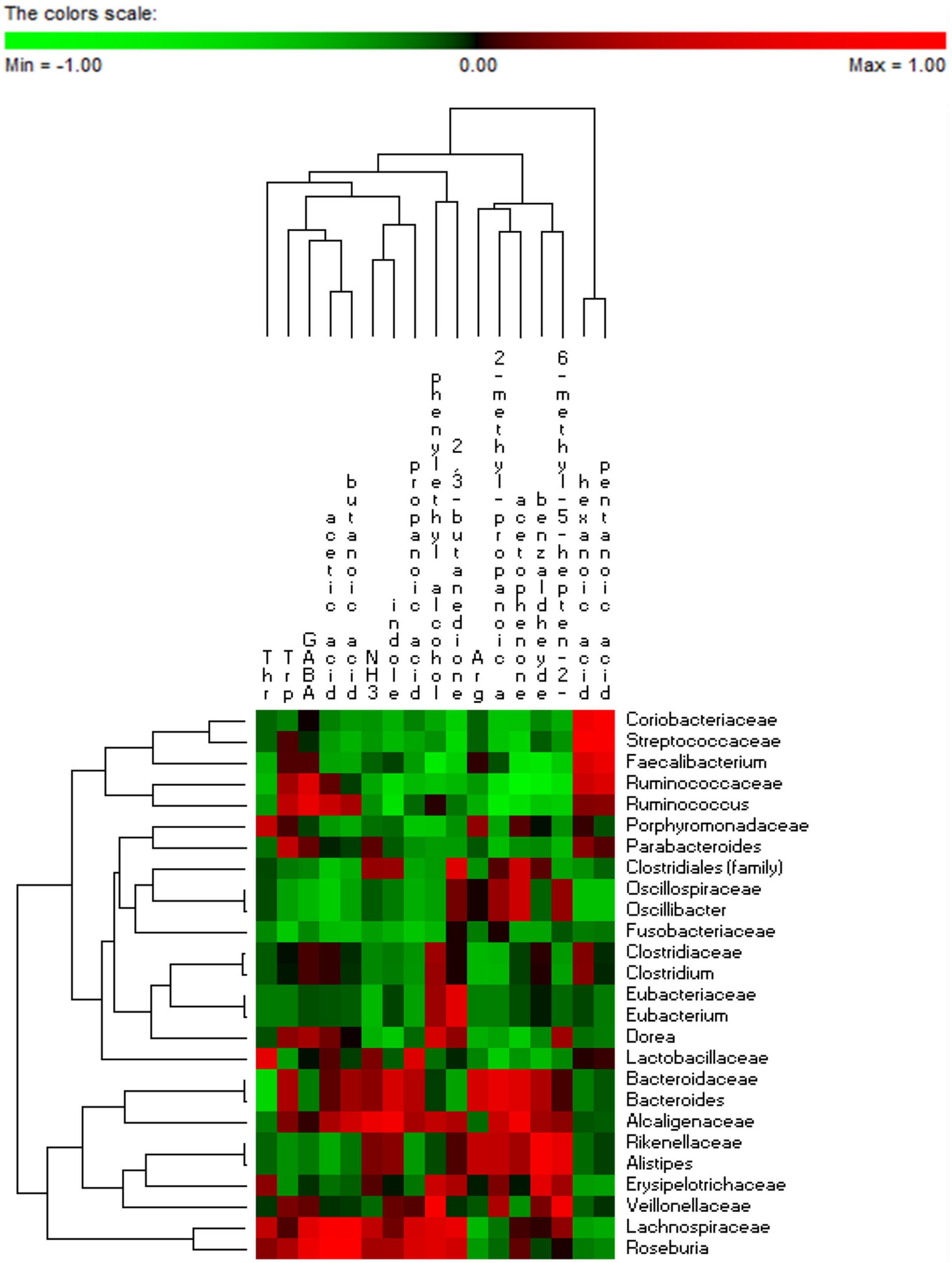


**FIG 6** Significant correlations between metabolically active bacterial OTUs (family and genus levels) and free amino acids and volatile organic compounds after 2 months of diet intervention with durum wheat flour and whole-grain barley pasta. The colors of the scale bar denote the nature of the correlation, with 1 indicating a perfectly positive correlation (red) and −1 indicating a perfectly negative correlation (green) between two microbial families or genera. Only significant correlations (FDR, <0.05) are shown. GABA, γ-aminobutyric acid.

Downloaded from http://aem.asm.org/ on October 22, 2015 by ALBERT EINSTEIN COLL OF MED

*aceae* (*Clostridium orbiscindens* and *Clostridium* sp.), *Roseburia hominis*, and *Ruminococcus* sp. increased following the intervention. Previously, it was shown that barley and oat β-glucans induced clostridial cluster IX populations and the *Clostridium histolyticum* subgroup during *in vitro* fermentation by human fecal microbiota (48). On the contrary, oat β-glucans did not favor the growth of *Clostridium* sp. in SHIME and C57BL/6J mouse models (21, 24, 49). However, several discrepancies between the different studies might be due to the different models and methods used. Levels of *F. prausnitzii*, *Faecalibacterium* sp., and *D. invisus* decreased following the diet intervention with barley β-glucans. A similar trend was found for members of the genus *Fusobacterium*, belonging to *Fusobacteria*. Based on OTU correlations, β-glucans negatively impacted bacterial interactions.

The positive bacterial interactions also decreased during dietary supplementation with β-glucans from sea cucumber (44). In agreement, the community-level catabolic profiles showed decreased substrate utilization pattern ($H'$ index) and Shannon index after diet intervention with barley β-glucans. All these results indicated low metabolic diversity. Culture-dependent methods showed that diet intervention with whole-grain barley markedly decreased the total number of fecal anaerobic cultivable bacteria. Decreased levels of cultivable anaerobes were also found during *in vitro* fermentation of oat β-glucans by human fecal slurry (20). According to the *in vitro* study conducted by Hughes et al. (48), levels of cultivable presumptive thermophilic and mesophilic *Lactobacillus* spp. increased following the diet intervention with barley β-glucans. No positive effects were observed regarding organisms of the genus *Bifidobacterium*. A randomized, placebo-controlled, and double-blind human intervention trial showed that cultivable fecal lactobacilli isolated from 26 healthy volunteers were not affected by 0.75 g of barley β-glucans (26). The same study showed a statistically significant increase in the cell density of bifidobacteria. After grouping volunteers according to age, a significant bifidogenic effect was detected only in subjects >50 years. The administration of 3 g/day of β-glucans to 33 polypectomized patients showed no effects on the fecal cultivable lactobacilli and bifidobacteria (27). An increased level of lactobacilli was found in animal models after a barley- or oat-containing diet, especially using high-viscosity β-glucans (22, 23, 24). At the same time, an oat product-based diet or β-glucans from *Laminaria digitata*, *Laminaria hyperborea*, and *Saccharomyces cerevisiae* did not increase the levels of lactobacilli in pigs and rats (25, 50). Taken together, these results suggested that the prebiotic potential of whole-grain barley/β-glucan was not always reproducible *in vivo*.

The complex biochemical interactions and antagonistic activities within the intestinal microbiota prior to treatment might be responsible for the different responses to the β-glucan-enriched diet. Levels of presumptive cultivable *Bacteroides*, *Porphyromonas*, and *Prevotella* spp. decreased following the diet intervention with barley β-glucans. Overall, *Prevotella* abundance is higher in humans who consume more of a vegetable-based diet (14). During *in vitro* fermentation, *Prevotella* and *Bacteroides* levels increased with low-molecular-weight β-glucans only (48). Levels of cultivable *Bacteroides* sp. decreased in the SHIME model after oat bran feeding (21), while no significant differences were found in rats after feeding with products enriched with oat fiber (50). A barley-rich diet was associated with a reduced level of cultivable *Bacteroides* sp. in the intestinal tract of rats (22). According to this study, cultivable *Bacteroides* was negatively affected in polypectomized patients after intake of barley β-glucans (27). *In vitro* and animal trials support that *Enterobacteriaceae* could not utilize β-glucans (20, 25, 50). According to *in vitro* and animal trials, this study showed a significant reduction in cultivable *Enterobacteriaceae* and total coliforms. A similar trend was found in the administration of 3 g/day of β-glucans to 33 polypectomized patients (27). On the contrary, small amounts of β-glucans (0.75 g) were not effective in decreasing the level of total cultivable coliforms or *E. coli* (26). First, this study showed that the cell density of cultivable *Pseudomonas*, *Alcaligenes*, and *Aeromonas* markedly decreased after the diet intervention with pasta enriched with barley β-glucans.

*In vitro* and animal trials support that high barley β-glucan consumption is associated with a significant increase in the synthesis of SCFA (46, 49, 50). This study confirmed the previous GC-MS data showing a noticeable increase in 2-methyl-propanoic acid, acetic acid, butanoic (butyric) acid, and propanoic (propionic) acid after the diet intervention with durum wheat flour and whole-grain barley pasta. SCFA induce positive gastrointestinal and systemic effects (51). SCFA are also important modulators of the host immune function (52). Acetate is used as an energy source for liver and peripheral tissues and acts as a signaling molecule in gluconeogenesis and lipogenesis (53). Propionate serves as a precursor for gluconeogenesis and reduces the synthesis of hepatic cholesterol (54). Propionate was also associated with a decrease in insulin secretion in pancreatic islet cells of rats (55). Butyric acid serves as the main energy source for colonocytes and protects against inflammation (56, 57). Butyrate also affects the regulation of apoptosis and cellular proliferation, resulting in a reduced risk of colon cancer (58). BCFA (e.g., isobutyrate and isovalerate) were not affected by whole-grain barley, indicating that the greatest synthesis of SCFA was obtained without increasing undesirable protein fermentation (44). Previously, it was demonstrated that the intake of dietary fibers or symbiotic foods (e.g., fructooligosaccharides, *Lactobacillus helveticus*, and *Bifidobacterium longum*) leads to a modulation of the gut metabolic activity, with an increase of SCFA (59–61). Previously, *in vitro* data showed that barley and oat β-glucans or other oligosaccharides support the growth of *Clostridia* strains, which synthesize acetate and butyrate (62, 63). *Bacteroides* strains are propionate producers via the succinate pathway (64), and they were positively correlated with propionic acid.

This study highlights some *in vivo* effects of whole-grain barley on the fecal microbiota and metabolome. Whole-grain barley appeared to be effective in the modulation of the composition and the metabolic pathways of the intestinal microbiota, leading to an increased level of SCFA.

## ACKNOWLEDGMENTS

This work was funded by the Ministero dell'Istruzione, dell'Università e della Ricerca (MIUR), Ministero dello Sviluppo Economico, and Fondo Europeo di Sviluppo Regionale (P.O.N. Ricerca e Competitività 2007–2013 per le Regioni della Convergenza, project code PON02_00657_00186_2937475/1; PROALIFUN), ASSE I – Sostegno ai Mutamenti Strutturali, Obiettivo Operativo "Reti per il rafforzamento del potenziale scientifico-tecnologico delle Regioni della Convergenza," azione I: "Distretti di Alta Tecnologia e relative Reti".

We thank Maria Piccolo (Department of Soil, Plant and Food Sciences, University of Bari Aldo Moro, Bari, Italy) and Giuseppe Dalfino and Stella Diamante (DETO, Nephrology Unit, University of Bari Aldo Moro, Bari, Italy) for technical assistance.

We declare no conflicts of interest.

Downloaded from http://aem.asm.org/ on October 22, 2015 by ALBERT EINSTEIN COLL OF MED

## REFERENCES


1. **Food and Drug Administration, Department of Health and Human Services.** 2005. Food labeling: health claims; soluble dietary fiber from certain foods and coronary heart disease. Interim final rule. Fed Regist **70:**76150–76162.
2. **Food and Drug Administration, Department of Health and Human Services.** 2008. Food labeling: health claims; soluble dietary fiber from certain foods and coronary heart disease. Interim final rule. Fed Regist **73:**9938–9947.
3. **European Food Safety Authority (EFSA).** 2010. Scientific opinion on dietary reference values for carbohydrates and dietary fibre. EFSA J **8:**1462.
4. **Tosh SM.** 2013. Review of human studies investigating the post-prandial blood-glucose lowering ability of oat and barley food products. Eur J Clin Nutr **67:**310–317. http://dx.doi.org/10.1038/ejcn.2013.25.
5. **Cloetens L, Ulmius M, Johansson-Persson A, Akesson B, Onning G.** 2012. Role of dietary beta-glucans in the prevention of the metabolic syndrome. Nutr Rev **70:**444–458. http://dx.doi.org/10.1111/j.1753-4887.2012.00494.x.
6. **Brown L, Rosner B, Willett WW, Sacks FM.** 1999. Cholesterol-lowering effects of dietary fiber: a meta-analysis. Am J Clin Nutr **69:**30–42.
7. **Bourdon I, Yokoyama W, Davis P, Hudson C, Backus R, Richter D, Knuckles B, Schneeman BO.** 1999. Postprandial lipid, glucose, insulin, and cholecystokinin responses in men fed barley pasta enriched with β-glucan. Am J Clin Nutr **69:**55–63.
8. **Lia A, Hallmans G, Sandberg AS, Sundberg B, Aman P, Andersson H.** 1995. Oat glucan increases bile acid excretion and a fiber-rich barley fraction increases cholesterol excretion in ileostomy subjects. Am J Clin Nutr **62:**1245–1251.
9. **Glore SR, Van Treeck D, Knehans AW, Guild M.** 1994. Soluble fiber and serum lipids: a literature review. J Am Diet Assoc **94:**425–436. http://dx.doi.org/10.1016/0002-8223(94)90099-X.
10. **Bell S, Goldman VM, Bistrian BR, Arnold AH, Ostroff G, Forse RA.** 1999. Effect of beta-glucan from oats and yeast on serum lipids. Crit Rev Food Sci Nutr **39:**189–202. http://dx.doi.org/10.1080/10408399908500493.
11. **Anderson JW, Deakins DA, Floore TL, Smith BM, Whitis SE.** 1990. Dietary fiber and coronary heart disease. Crit Rev Food Sci Nutr **29:**95–147. http://dx.doi.org/10.1080/10408399009527518.
12. **Karlsson F, Tremaroli V, Nielsen J, Bäckhed F.** 2013. Assessing the human gut microbiota in metabolic diseases. Diabetes **62:**3341–3349. http://dx.doi.org/10.2337/db13-0844.
13. **Zhang PP, Hu XZ, Zhen HM, Xu C, Fan MT.** 2012. Oat β-glucan increased ATPases activity and energy charge in small intestine of rats. J Agric Food Chem **60:**9822–9827. http://dx.doi.org/10.1021/jf3017496.
14. **Montemurno E, Cosola C, Dalfino G, Daidone G, De Angelis M, Gobbetti M, Gesualdo L.** 2014. What would you like to eat, Mr CKD microbiota? A Mediterranean diet, please! Kidney Blood Press Res **39:**114–123. http://dx.doi.org/10.1159/000355785.
15. **Flint HJ, Duncan SH, Scott KP, Louis P.** 2007. Interactions and competition within the microbial community of the human colon: links between diet and health. Environ Microbiol **9:**1101–1111. http://dx.doi.org/10.1111/j.1462-2920.2007.01281.x.
16. **Bergman EN.** 1990. Energy contributions of volatile fatty acids from the gastrointestinal tract in various species. Physiol Rev **70:**567–590.
17. **Bernalier-Donadille A.** 2012. Fermentative metabolism by the human gut microbiota. Gastroenterol Clin Biol **34**(Suppl 1)**:**S16–S22. (In French.)
18. **Bäckhed F, Hao D, Wang T, Hooper LV, Koh GY, Nagy A, Semenkovich CF, Gordon JI.** 2004. The gut microbiota as an environmental factor that regulates fat storage. Proc Natl Acad Sci U S A **101:**15718–15723. http://dx.doi.org/10.1073/pnas.0407076101.
19. **Bindels LB, Delzenne NM, Cani PD, Walter J.** 2015. Towards a more comprehensive concept for prebiotics. Nat Rev Gastroenterol Hepatol **12:**303–310. http://dx.doi.org/10.1038/nrgastro.2015.47.
20. **Jaskari J, Kontula P, Siitonen A, Jousimies-Somer H, Mattila-Sandholm T, Poutanen K.** 1998. Oat β-glucan and xylan hydrolysates as selective substrates for *Bifidobacterium* and *Lactobacillus* strains. Appl Microbiol Biotechnol **49:**175–181. http://dx.doi.org/10.1007/s002530051155.
21. **Kontula P, Jaskari J, Nollet L, De Smet I, von Wright A, Poutanen K, Mattila-Sandholm T.** 1998. The colonization of a simulator of the human intestinal microbial ecosystem by a probiotic strain fed on a fermented oat bran product effects on the gastrointestinal microbiota. Appl Microbiol Biotechnol **50:**246–252. http://dx.doi.org/10.1007/s002530051284.
22. **Dongowski G, Huth M, Gebhardt E, Flamme W.** 2002. Dietary fiber-rich barley products beneficially affect the intestinal tract of rats. J Nutr **132:**3704–3714.
23. **Snart J, Bibiloni R, Grayson T, Lay C, Zhang H, Allison GE, Laverdiere JK, Temelli F, Vasanthan T, Bell R, Tannock GW.** 2006. Supplementation of the diet with high-viscosity beta-glucan results in enrichment for lactobacilli in the rat cecum. Appl Environ Microbiol **72:**1925–1931. http://dx.doi.org/10.1128/AEM.72.3.1925-1931.2006.
24. **Zhou AL, Hergert N, Rompato G, Lefevre M.** 2014. Whole grain oats improve insulin sensitivity and plasma cholesterol profile and modify gut microbiota composition in C57BL/6J mice. J Nutr **145:**222–230.
25. **Murphy P, Dal Bello F, O'Doherty J, Arendt EK, Sweeney T, Coffey A.** 2013. Analysis of bacterial community shifts in the gastrointestinal tract of pigs fed diets supplemented with β-glucan from *Laminaria digitata*, *Laminaria hyperborea* and *Saccharomyces cerevisiae*. Animal **7:**1079–1087. http://dx.doi.org/10.1017/S1751731113000165.
26. **Mitsou EK, Panopoulou N, Turunen K, Spiliotis V, Kyriacou A.** 2010. Prebiotic potential of barley derived β-glucan at low intake levels: a randomised, double-blinded, placebo-controlled clinical study. Food Res Int **43:**1086–1092. http://dx.doi.org/10.1016/j.foodres.2010.01.020.
27. **Turunen K, Tsouvelakidou E, Nomikos T, Mountzouris KC, Karamanolis D, Triantafillidis J, Kyriacou A.** 2011. Impact of beta-glucan on the fecal microbiota of polypectomized patients: a pilot study. Anaerobe **17:**403–406. http://dx.doi.org/10.1016/j.anaerobe.2011.03.025.
28. **Friedewald WT, Levy RI, Fredrickson DS.** 1972. Estimation of the concentration of low-density lipoprotein cholesterol in plasma, without use of the preparative ultracentrifuge. Clin Chem **18:**499–502.
29. **Gowen CM, Fong SS.** 2010. Genome-scale metabolic model integrated with RNAseq data to identify metabolic states of *Clostridium thermocellum*. Biotechnol J **5:**759–767.
30. **Suchodolski JS, Dowd SE, Wilke V, Steiner JM, Jergens AE.** 2012. 16S rRNA gene pyrosequencing reveals bacterial dysbiosis in the duodenum of dogs with idiopathic inflammatory bowel disease. PLoS One **7:**e39333. http://dx.doi.org/10.1371/journal.pone.0039333.
31. **Gontcharova V, Youn E, Wolcott RD, Hollister EB, Gentry TJ, Dowd SE.** 2010. Black box chimera check (B2C2): a Windows-based software for batch depletion of chimeras from bacterial 16S rRNA gene datasets. Open Microbiol J **4:**47–52. http://dx.doi.org/10.2174/1874285801004010047.
32. **Dowd SE, Zaragoza J, Rodriguez JR, Oliver MJ, Payton PR.** 2005. Windows. NET network distributed Basic Local Alignment Search Toolkit (W.ND-BLAST). BMC Bioinformatics **6:**93.
33. **Edgar RC.** 2010. Search and clustering orders of magnitude faster than BLAST. Bioinformatics **26:**2460–2461. http://dx.doi.org/10.1093/bioinformatics/btq461.
34. **Cole JR, Wang Q, Cardenas E, Fish J, Chai B, Farris RJ, Kulam-Syed-Mohideen AS, McGarrell DM, Marsh T, Garrity GM, Tiedje JM.** 2009. The Ribosomal Database Project: improved alignments and new tools for rRNA analysis. Nucleic Acids Res **37:**D141–D145.
35. **Oksanen J, Blanchet FG, Kindt R, Legendre P, O'Hara RB, Simpson GL, Solymos P, Stevens MHH, Wagner H.** 2011. vegan: community ecology package, R package version 1.17-1. https://cran.r-project.org/web/packages/vegan/index.html.
36. **Roberts DW.** 2010. labdsv: ordination and multivariate analysis for ecology. R package version 1.4-1. https://cran.r-project.org/web/packages/labdsv/.
37. **Anders S, Huber W.** 2010. Differential expression analysis for sequence count data. Genome Biol **11:**R106. http://dx.doi.org/10.1186/gb-2010-11-2-106.
38. **McMurdie PJ, Holmes S.** 2013. phyloseq: an R package for reproducible interactive analysis and graphics of microbiome census data. PLoS One **8:**e61217. http://dx.doi.org/10.1371/journal.pone.0061217.
39. **Crecchio C, Gelsomino A, Ambrosoli R, Minatic JL, Ruggiero P.** 2004. Functional and molecular responses of soil microbial communities under differing soil management practices. Soil Biol Biochem **36:**1873–1883. http://dx.doi.org/10.1016/j.soilbio.2004.05.008.
40. **Zak JC, Willing MR, Moorhead DL, Wildman HG.** 1994. Functional diversity of microbial communities a quantitative approach. Soil Biol Biochem **26:**1101–1108. http://dx.doi.org/10.1016/0038-0717(94)90131-7.
41. **De Angelis M, Piccolo M, Vannini L, Siragusa S, De Giacomo A, Siragusa S, Serrazzanetti DI, Cristofori F, Guerzoni ME, Gobbetti M, Francavilla R.** 2013. Fecal microbiota and metabolome of children with autism and pervasive developmental disorder not otherwise specified. PLoS One **8:**e76993. http://dx.doi.org/10.1371/journal.pone.0076993.
42. **Ndagijimana M, Laghi L, Vitali B, Placucci G, Brigidi P, Guerzoni ME.** 2009. Effect of a synbiotic food consumption on human gut metabolic


Downloaded from http://aem.asm.org/ on October 22, 2015 by ALBERT EINSTEIN COLL OF MED

profiles evaluated by $^{1}$H nuclear magnetic resonance spectroscopy. Int J Food Microbiol **134:**147–153.
43. **De Filippis F, Vannini L, La Storia A, Laghi L, Piombino P, Stellato G, Serrazanetti DI, Gozzi G, Turroni S, Ferrocino I, Lazzi C, Di Cagno R, Gobbetti M, Ercolini D.** 2014. The same microbiota and a potentially discriminant metabolome in the saliva of omnivore, ovo-lactovegetarian and vegan individuals. PLoS One **9:**e112373. http://dx.doi.org/10.1371/journal.pone.0112373.
44. **Yang J, Xu Z, Tian X, Dong S, Peng M.** 2015. Intestinal microbiota and immune related genes in sea cucumber (*Apostichopus japonicus*) response to dietary β-glucan. Biochem Biophys Res Commun **458:**98–103.
45. **Kühlwein H, Merrifield DL, Rawling MD, Foey AD, Davies SJ.** 2014. Effects of dietary β-(1,3)(1,6)-D-glucan supplementation on growth performance, intestinal morphology and haemato-immunological profile of mirror carp (*Cyprinus carpio* L). J Anim Physiol Anim Nutr (Berl) **98:**279–289.
46. **Yang J, Martínez I, Walter J, Keshavarzian A, Rose DJ.** 2013. *In vitro* characterization of the impact of selected dietary fibers on fecal microbiota composition. Anaerobe **23:**74–81. http://dx.doi.org/10.1016/j.anaerobe.2013.06.012.
47. **Greiner T, Bäckhed F.** 2011. Effects of the gut microbiota on obesity and glucose homeostasis. Trends Endocrinol Metab **22:**117–123. http://dx.doi.org/10.1016/j.tem.2011.01.002.
48. **Hughes SA, Shewry PR, Gibson GR, McCleary BV, Rastall RA.** 2008. *In vitro* fermentation of oat and barley derived β-glucans by human fecal microbiota. FEMS Microbiol Ecol **64:**482–493. http://dx.doi.org/10.1111/j.1574-6941.2008.00478.x.
49. **Shimizu J, Tsuchihashi N, Kudoh K, Wada M, Takita T, Innami S.** 2001. Dietary curdlan increases proliferation of bifidobacteria in the cecum of rats. Biosci Biotechnol Biochem **65:**466–469. http://dx.doi.org/10.1271/bbb.65.466.
50. **Drzikova B, Dongowski G, Gebhardt E.** 2005. Dietary fibre-rich oat-based products affect serum lipids, microbiota, formation of short-chain fatty acids and steroids in rats. Br J Nutr **94:**1012–1025. http://dx.doi.org/10.1079/BJN20051577.
51. **Wong JM, de Souza R, Kendall CW, Emam A, Jenkins DJ.** 2006. Colonic health fermentation and short chain fatty acids. J Clin Gastroenterol **40:**235–243. http://dx.doi.org/10.1097/00004836-200603000-00015.
52. **Tilg H, Moschen AR.** 2014. Microbiota and diabetes: an evolving relationship. Gut **63:**1513–1521. http://dx.doi.org/10.1136/gutjnl-2014-306928.
53. **Zambell KL, Fitch MD, Fleming SE.** 2003. Acetate and butyrate are the major substrates for *de novo* lipogenesis in rat colonic epithelial cells. J Nutr **133:**3509–3515.
54. **Cheng HH, Lai MH.** 2000. Fermentation of resistant rice starch produces propionate reducing serum and hepatic cholesterol in rats. J Nutr **130:**1991–1995.
55. **Ximenes H, Hirata AE, Rocha MS, Curi R, Carpinelli AR.** 2007. Propionate inhibits glucose-induced insulin secretion in isolated rat pancreatic islets. Cell Biochem Funct **25:**173–178. http://dx.doi.org/10.1002/cbf.1297.
56. **Ogawa H, Iimura M, Eckmann L, Kagnoff MF.** 2004. Regulated production of the chemokine CCL28 in human colon epithelium. Am J Physiol Gastrointest Liver Physiol **287:**G1062–G1069. http://dx.doi.org/10.1152/ajpgi.00162.2004.
57. **Canani RB, Di Costanzo M, Leone L, Pedata M, Meli R, Calignano A.** 2011. Potential beneficial effects of butyrate in intestinal and extraintestinal diseases. World J Gastroenterol **17:**1519–1528. http://dx.doi.org/10.3748/wjg.v17.i12.1519.
58. **Scheppach W, Bartram H, Richter F.** 1995. Role of short-chain fatty acids in the prevention of colorectal cancer. Eur J Cancer **31A:**1077–1080. http://dx.doi.org/10.1016/0959-8049(95)00165-F.
59. **Vitali B, Ndagijimana M, Cruciani F, Carnevali P, Candela M, Guerzoni ME, Brigidi P.** 2010. Impact of a synbiotic food on the gut microbial ecology and metabolic profiles. BMC Microbiol **10:**4. http://dx.doi.org/10.1186/1471-2180-10-4.
60. **Candela M, Maccaferri S, Turroni S, Carnevali P, Brigidi P.** 2010. Functional intestinal microbiome, new frontiers in prebiotic design. Int J Food Microbiol **140:**93–101. http://dx.doi.org/10.1016/j.ijfoodmicro.2010.04.017.
61. **Candela M, Biagi E, Turroni S, Maccaferri S, Figini P, Brigidi P.** 2015. Dynamic efficiency of the human intestinal microbiota. Crit Rev Microbiol **41:**165–171. http://dx.doi.org/10.3109/1040841X.2013.813900.
62. **Mitsuoka T.** 1996. Intestinal flora and human health. Asia Pac J Clin Nutr **5:**2–9.
63. **Shimizu T, Ohtani K, Hirakawa H, Ohshima K, Yamashita A, Shiba T, Ogasawara N, Hattori M, Kuhara S, Hayashi H.** 2002. Complete genome sequence of *Clostridium perfringens*, an anaerobic flesh-eater. Proc Natl Acad Sci U S A **99:**996–1001. http://dx.doi.org/10.1073/pnas.022493799.
64. **Louis P, Scott KP, Duncan SH, Flint HJ.** 2007. Understanding the effects of diet on bacterial metabolism in the large intestine. J Appl Microbiol **102:**1197–1208. http://dx.doi.org/10.1111/j.1365-2672.2007.03322.x.

Downloaded from http://aem.asm.org/ on October 22, 2015 by ALBERT EINSTEIN COLL OF MED
