# Supplementary material for: Beta-Glucans Supplementation Associates with Reduction in P-Cresyl Sulfate Levels and Improved Endothelial Vascular Reactivity in Healthy Individuals
Source: PLoS One. 2017 Jan 20;12(1):e0169635. doi: 10.1371/journal.pone.0169635 (PMC5249102; doi:10.1371/journal.pone.0169635)
Supplement: S2 File — Original study protocol as approved by the local Ethics Committee (document in Italian). (DOCX) [file pone.0169635.s004.docx]

**PROTOCOLLO CLINICO**

**TITOLO DEL PROGETTO**

PROTOCOLLI TECNOLOGICI E CLINICI INNOVATIVI PER LA PRODUZIONE DI ALIMENTI FUNZIONALI (Pro.Ali.Fun.)

**SOGGETTO ATTUATORE E FINANZIAMENTO**

Distretto Agroalimentare Regionale - D.A.Re. scarl

Finanziamento: fondi PON R&C 2007-2013 - ASSE I “Sostegno ai mutamenti strutturali” - OBIETTIVO OPERATIVO I.3 ”Reti per il rafforzamento del potenziale scientifico-tecnologico delle Regioni della Convergenza” di cui all’Avviso DD n.713/Ric. del 29 ottobre 2010, ai sensi dell’art. 13 del DM 593/2000, e del relativo decreto di concessione n.648/RIC del 08 ottobre 2012.

**OBIETTIVO SPERIMENTALE**

OS 6.5 Valutazione dell’efficacia clinica di una pasta arricchita in beta-glucani di orzo e avena su soggetti affetti da sindrome metabolica

**SCOPO DELLO STUDIO CLINICO**

Lo scopo del presente studio clinico è valutare le proprietà salutistiche di un intervento dietetico-nutrizionale condotto con una innovativa pasta arricchita con fibre prebiotiche (beta-glucani di orzo e avena).

L’ipotesi è che il microbiota intestinale e metaboloma, lo stato nutrizionale, redox, di infiammazione sub-clinica ed i markers di rischio cardiovascolare possano migliorare in soggetti sani sottoposti ad una dieta con pasta arricchita con fibre prebiotiche.

In particolare, i risultati dello studio su tale popolazione di soggetti sani andrà a corroborare i risultati di uno studio parallelo, condotto dall’Università di Foggia nell’ambito dello stesso progetto Pro.Ali.Fun., su soggetti con sindrome metabolica.

A tal fine, si procederà all’arruolamento di un gruppo di soggetti sani, funzionale alla dimostrazione dell’ipotesi proposta.

**CARATTERISTICHE DELLO STUDIO CLINICO**

Il presente trial clinico è uno studio pilota prospettico.

Esso avrà una durata di 4 mesi e verrà condotto presso il Dipartimento DETO – Sezione di Nefrologia, Dialisi e Trapianti dell’Università degli Studi di Bari “Aldo Moro”.

Saranno inclusi nello studio 40 soggetti sani (suddivisi in un due gruppi: un gruppo di 10 soggetti controllo + un gruppo di 30 soggetti da sottoporre al trial), che rispondano ai criteri di inclusione ed esclusione, valutati al momento dell’arruolamento, e che abbiano firmato il consenso informato.

**CRITERI DI INCLUSIONE ED ESCLUSIONE**

Verranno reclutati i pazienti che rispettino i seguenti criteri di inclusione ed esclusione:

***Criteri di inclusione***: Soggetti sani, di età compresa tra i 30 ed i 70 anni; BMI compreso fra 18,5 e 24,9; dieta onnivora; firma del consenso informato.

***Criteri di esclusione***: Familiarità con diabete mellito di tipo 2, utilizzo di antibiotici o di probiotici fino a 15 gg prima del reclutamento, patologie gastrointestinali, proteinuria superiore a 1 g/die, morbo celiaco, patologie infiammatorie sistemiche, sospetto o diagnosi clinica di neoplasie, epatopatia cronica, terapie con corticosteroidi o farmaci immunosoppressori, precedenti patologie acute cardiocircolatorie maggiori (infarto del miocardio, ictus), condizioni psichiatriche che riducano la compliance ai protocolli terapeutici.

**SOGGETTI CONTROLLO**

10 soggetti (5 maschi e 5 femmine) eleggibili secondo i criteri di inclusione ed esclusione verranno reclutati come controlli sani dello studio e verrà chiesto loro di fornire campioni biologici (feci, saliva, urine, sangue). Tali campioni verranno utilizzati per la messa a punto delle metodiche che verranno successivamente utilizzate per l’analisi dei campioni raccolti durante il trial clinico.

**SOGGETTI DA SOTTOPORRE AL TRIAL E MODALITÀ DI RECLUTAMENTO**

30 soggetti eleggibili secondo i criteri di inclusione ed esclusione verranno reclutati e verrà loro prescritto un regime dietetico controllato (secondo le linee guida INRAN) per due mesi. Nei successivi due mesi, nell’ambito dello stesso regime controllato, verrà introdotta la somministrazione di una porzione giornaliera di pasta con beta-glucani.

**RACCOLTA DATI E CAMPIONI BIOLOGICI**

Ciascun paziente verrà seguito ad intervalli di due mesi. Tutti i dati sperimentali ottenuti, insieme con i principali dati anagrafici di ciascun paziente saranno riportati in una Case Report Form (CRF) elettronica. Al reclutamento, T0 e T2 mesi dall’inizio del trial verranno raccolti i seguenti dati e campioni biologici:

- Dati anagrafici
- Informazioni relative alle abitudini alimentari (FFQ Food Frequency Questionnaire e diario alimentare 24h)
- Dati bioimpedenziometrici
- Pressione arteriosa
- Flow-mediated dilation (FMD - misura della vasodilatazione endotelio-mediata di un’arteria sottoposta ad incremento di flusso e a stress meccanico)
- Stiffness arteriosa (baPWV – velocità di propagazione dell’onda sfigmica)
- Campioni di sangue
- Campioni di urine
- Campioni di feci

**ANALISI ED INFORMAZIONI RACCOLTE DAI CAMPIONI BIOLOGICI**

I campioni biologici raccolti saranno funzionali alla determinazione dei seguenti parametri:

- sodiemia e sodiuria
- potassiemia
- cloremia
- creatinina urinaria
- microalbuminuria
- glicemia e HbA1c
- C-peptide
- trigliceridi, colesterolo totale, colesterolo LDL e colesterolo HDL
- profilo emocoagulativo
- VES e proteina C reattiva (PCR) ad alta sensibilità
- fibrinogeno
- uricemia, azotemia, creatininemia
- AST, ALT, gammaGT, fosfatasi alcalina, CPK
- proteine totali con elettroforesi, emocromo, bilirubina totale
- esame urine completo
- calcemia, fosforemia, sideremia, ferritina, transferrina
- PTH e vitamina D
- 8-oxo-dG sierico e oxLDL
- Identificazione e caratterizzazione dei principali gruppi microbici presenti nei campioni fecali e metaboloma (SCFA, pCS, IS)

**ANALISI STATISTICA**

Tutte le analisi saranno condotte sulla base dell’“Intention to treat”. Le variabili dicotomiche o policotomiche saranno analizzate mediante il Fisher exact test. Le variabili continue saranno invece analizzate mediante l’ANCOVA utilizzando i valori basali come covariate.

Lo Sperimentatore principale

___________________________________________
