# Supplementary material for: Beta-Glucans Supplementation Associates with Reduction in P-Cresyl Sulfate Levels and Improved Endothelial Vascular Reactivity in Healthy Individuals
Source: PLoS One. 2017 Jan 20;12(1):e0169635. doi: 10.1371/journal.pone.0169635 (PMC5249102; doi:10.1371/journal.pone.0169635)
Supplement: S3 File — English translation of the study protocol. (DOCX) [file pone.0169635.s005.docx]

**CLINICAL PROTOCOL**

**PROJECT TITLE**

TECHNOLOGICAL AND CLINICAL INNOVATIVE PROTOCOLS FOR THE PRODUCTION OF FUNCTIONAL FOODS - 6.5 (ProAliFun65)

**LEAD PARTNER AND FUNDING SOURCE**

LEAD PARTNER: Distretto Agroalimentare Regionale - D.A.Re. scarl

FUNDING: fondi PON R&C 2007-2013 - ASSE I “Sostegno ai mutamenti strutturali” - OBIETTIVO OPERATIVO I.3 ”Reti per il rafforzamento del potenziale scientifico-tecnologico delle Regioni della Convergenza” di cui all’Avviso DD n.713/Ric. del 29 ottobre 2010, ai sensi dell’art. 13 del DM 593/2000, e del relativo decreto di concessione n.648/RIC del 08 ottobre 2012.

**AIM OF THE STUDY**

The purpose of this study is to evaluate the healthy properties of a dietary intervention based on an innovative pasta enriched with prebiotic fibers (barley beta-glucans).

The hypothesis is that the gut microbiota and metabolome, the nutritional status, the redox/subclinical inflammation parameters and the markers of cardiovascular risk may improve in healthy subjects.

**CHARATERISTICS OF THE CLINICAL TRIAL**

This is a pilot interventional, open label, single-group assignment study.

It will last 4 months and will be carried on at the ambulatories of the Nephrology and Cardiovascular Disease Units of the DETO – Department of Emergency and Organ Transplantation of the University of Bari “Aldo Moro”.

A target number of 30 healthy individuals meeting inclusion and exclusion criteria, assessed at enrolment, and which have signed the informed consent, will be recruited.

**INCLUSION AND EXCLUSION CRITERIA**

Healthy volunteers will be enrolled according to the following inclusion/exclusion criteria:

**Inclusion criteria:**

- healthy people aged 30-70 years old
- BMI ranged between 18.5 and 24.9
- omnivorous diet
- informed consent signed

**Exclusion criteria:**

- diabetes type 2
- urine protein>1g/24h
- antibiotics and probiotics administration by 15 days before the enrollment
- gastrointestinal, celiac, inflammatory systemic and chronic liver diseases
- recent diagnosis of cancer
- corticosteroid or immunosuppressive therapies
- previous major acute cardiovascular pathologies (heart attack, cerebral ictus)
- hyperlipidemia
- consume of alcohol
- psychiatric diseases

**STUDY DESIGN**

Thirty subjects eligible according to inclusion and exclusion criteria will be recruited and advised to follow a Mediterranean-based free diet (according to INRAN guidelines) including a daily supply of 100 gr of usual pasta for two months (run-in period). In the following two months, under the same dietary scheme, a daily portion of pasta with beta-glucans (“Cuore Mio” Granoro) will be introduced.

**DATA COLLECTION AND BIOLOGICAL SAMPLES**

Each person will be visited every two months.

Before (T0) and after (T2) the 2 months of beta-glucans intervention, the following data/samples will be collected:

- Information relating to eating habits (FFQ Food Frequency Questionnaire and Food 24h diary)
- Bioimpedance data
- Blood pressure
- Flow-mediated dilation (FMD - measure of endothelium-mediated artery subjected to increased flow and mechanical stress)
- Arterial Stiffness (baPWV - pulse wave velocity)
- Blood samples
- Urine Samples
- Stool specimens

**ANALYES TO BE PERFORMED**

The biological samples will be analysed for the following determinations:

- Blood routine test
- Urine routine test
- Serum pCS, IS, 8-oxo-dG, ox-LDL
- Fecal microbiota and metabolome characterization

**STATISTICAL ANALYSIS**

All the analyses will be based on “intention to treat”. Dichotomous or policotomic variables will be analyzed by the Fisher exact test. Continuous variables will instead be analyzed by ANCOVA using baseline values ​​as covariates.
