# Supplementary material for: Beta-Glucans Supplementation Associates with Reduction in P-Cresyl Sulfate Levels and Improved Endothelial Vascular Reactivity in Healthy Individuals
Source: PLoS One. 2017 Jan 20;12(1):e0169635. doi: 10.1371/journal.pone.0169635 (PMC5249102; doi:10.1371/journal.pone.0169635)
Supplement: S4 File — Mediterranean-based dietary advices–translation of the dietary advice distributed by the volunteers at the beginning of the study. (DOCX) [file pone.0169635.s006.docx]

**MEDITERRANEAN-BASED DIETARY ADVICES**

In preparing your meals, please keep in consideration the following dietary advices:

- Eat at least 5 portions of vegetables and fresh fruit and increase the consumption of fresh and/or dried legumes
- Eat regularly bread, pasta (100 gr/day), rice or other grains (not whole)
- Limit the amounts of fats and oils used for seasoning and cooking and possibly replace them with herbs and spices
- Limit the intake of animal fats (butter, lard, cream, etc.), preferring especially extra-virgin olive oil (mainly raw)
- Eat more often fish, both fresh and frozen (2-3 times a week)
- Among the meats, prefer the white ones (2-3 times a week) and limit the consumption of red ones (monthly intake)
- Consume up to 4 eggs per week, distributed in several days
- Choose preferably skimmed or semi-skimmed milk
- All cheeses contain high amounts of fat: choose those leaner ones or consume smaller portions
- Moderate the consumption of sweet foods and drinks during the day, in order not to exceed the amount of sugar allowed
- Drink 1.5-2 liters of water per day
- Reduce the use of salt in the preparation of meals and limit the use of dressings containing sodium (stock cubes, ketchup, soy sauce, mustard, etc.)
- Drink alcohol in moderation, one glass of wine per meal for men and half for women is allowed
- Eat local, seasonal products, respecting the conviviality
- Practice a regular physical activity
- Avoid the use of probiotics/prebiotics and of whole grain cereals during the study period
